# Supplementary material for: RREB1 regulates neuronal proteostasis and the microtubule network
Source: Sci Adv. 2024 Jan 10;10(2):eadh3929. doi: 10.1126/sciadv.adh3929 (PMC10780896; doi:10.1126/sciadv.adh3929)
Supplement: 20240110-1 [file sciadv.adh3929.v1.pdf]

MOLECULAR BIOLOGY

# RREB1 regulates neuronal proteostasis and the microtubule network

**Emily N. Griffin[1], Thomas Jucius[1], Su-Eon Sim[1], Belinda S. Harris[2], Sven Heinz[3], Susan L. Ackerman[1]***

Copyright © 2024 The Authors, some rights reserved; exclusive licensee American Association for the Advancement of Science. No claim to original U.S. Government Works. Distributed under a Creative Commons Attribution License 4.0 (CC BY).

**Transcription factors play vital roles in neuron development; however, little is known about the role of these proteins in maintaining neuronal homeostasis. Here, we show that the transcription factor RREB1 (Ras-responsive element-binding protein 1) is essential for neuron survival in the mammalian brain. A spontaneous mouse mutation causing loss of a nervous system–enriched *Rreb1* transcript is associated with progressive loss of cerebellar Purkinje cells and ataxia. Analysis of chromatin immunoprecipitation and sequencing, along with RNA sequencing data revealed dysregulation of RREB1 targets associated with the microtubule cytoskeleton. In agreement with the known role of microtubules in dendritic development, dendritic complexity was disrupted in *Rreb1*-deficient neurons. Analysis of sequencing data also suggested that RREB1 plays a role in the endomembrane system. Mutant Purkinje cells had fewer numbers of autophagosomes and lysosomes and contained P62- and ubiquitin-positive inclusions. Together, these studies demonstrate that RREB1 functions to maintain the microtubule network and proteostasis in mammalian neurons.**

## INTRODUCTION

Although great diversity in cell specificity, molecular disruptions, and behavioral alterations exists among neurodegenerative disorders, protein inclusions are a unifying pathological hallmark associated with many of these diseases (*1*). The process of regulating proteins in the cell, or proteostasis, is maintained via two protein degradation mechanisms: autophagy and proteasome-mediated degradation. Defects in either autophagy or proteasome function have been associated with the formation of protein inclusions. Inclusion formation and subsequent cell death in sporadic forms of neurodegenerative diseases may be linked directly to dysfunction in proteostasis.

The role of transcription factors has long been examined in the context of cellular differentiation and multicellular organism growth and development; however, recent studies have extended the role of these proteins in regulation of cellular homeostasis in terminally differentiated cells. A few studies have highlighted transcription factors that function in neuron homeostasis and, more specifically, neuronal proteostasis. For example, the transcription factor EB (TFEB) (so named for its recognition and binding to E-box motifs) belongs to the family of microphthalmia/transcription factor E (*MiT/TFE*) genes, has been shown to control expression of genes involved in autophagosome formation and lysosome biogenesis, and has reduced expression in many neurodegenerative diseases (*2*–*4*). Another MiT/TFE transcription factor, transcription factor binding to immunoglobulin heavy constant mu enhancer 3, is reduced in neuronal nuclei of patients with Parkinson's disease and has recently been shown to positively regulate autophagy (*5*). Although these studies have illuminated a likely role for transcription factors in maintaining proteostasis in a pathological context, causal mutations in genes encoding these transcription factors have rarely been identified.

Our study demonstrates that hypomorphic loss of the transcription factor RREB1 is associated with the formation of protein inclusions in cerebellar Purkinje cells and subsequent neurodegeneration. Chromatin immunoprecipitation and sequencing (ChIP-seq) and RNA sequencing (RNA-seq) experiments suggest that RREB1 positively regulates the endomembrane system. We also find that RREB1 positively regulates many genes associated with the microtubule cytoskeletal network. Disruption of these genes is associated with changes to the microtubule network as well as impaired dendritic branching. Together, our data demonstrate a unique role for RREB1 in neuron homeostasis.

## RESULTS

### *Rreb1 V7* deficiency is associated with Purkinje cell loss

The new mutant 3888 (*nm3888*) mutation arose spontaneously in nonobese diabetic (NOD).129P2(Cg)-*Il10*$^{tm1Cgn}$/DvsJ mice and was backcrossed to NOD mice to remove the *Il10* mutation. Mice homozygous for the *nm3888* mutation had mild ataxia and tremors beginning at 4 weeks of age (movies S1 and S2). Immunohistochemistry for calbindin D-28, a calcium-binding protein expressed in Purkinje cells, revealed Purkinje cell degeneration beginning at 4 weeks of age (Fig. 1A). Death of these neurons progressed rostrally to caudally with approximately 90% survival at 1 month of age, 35% at 6 months of age, and 16% at 12 months of age (Fig. 1B). Purkinje cells in mice heterozygous for this mutation also degenerated, but cell death had a later onset beginning at 6 months of age, and death of these neurons progressed more slowly with cell survival at 77% at 3 months of age and 72% at 12 months of age. Cleaved caspase 3 was detected in dying neurons but not wild-type neurons, indicating that these cells were undergoing apoptosis (Fig. 1C).

Genome scans, using polymorphic microsatellite markers on F2 *nm3888*$^{-/-}$ mice generated from crosses of NOD/ShiLtJ-*nm3888*$^{-/-}$ and CAST/Ei mice, revealed the *nm3888* mutation was on Chromosome 13. Analysis of additional F2 mice demonstrated that the *nm3888* mutation resided in a 2-megabase (Mb) region between *D13Mit18* and *rs291434* on Chromosome 13, a region containing eight protein-coding genes (Fig. 2A). To examine the expression levels of protein-coding

[1]Howard Hughes Medical Institute, Department of Cellular and Molecular Medicine, Department of Neurobiology, University of California, San Diego, La Jolla, CA 92093, USA. [2]The Jackson Laboratory, Bar Harbor, ME 04609, USA. [3]Department of Medicine, University of California, San Diego, La Jolla, CA 92093, USA.
*Corresponding author. Email: sackerman@ucsd.edu

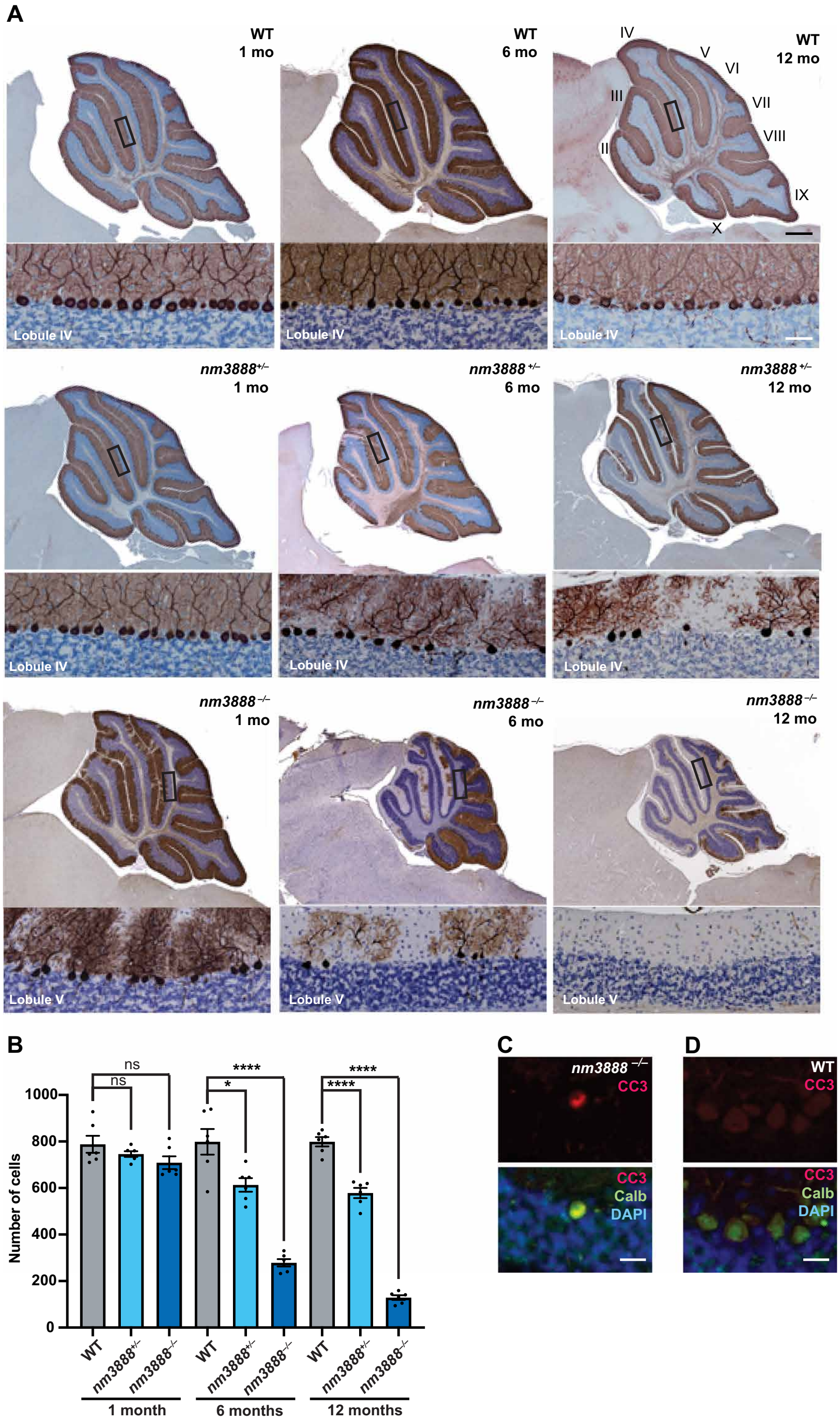


**Fig. 1. Purkinje cell degeneration occurs in *nm3888* mutant mice.** (**A**) Calbindin D-28 immunohistochemistry and hematoxylin staining of sagittal sections of cerebella from *nm3888*$^{-/-}$, *nm3888*$^{+/-}$, and wild-type (WT) mice at ages indicated. High-magnification images of boxed regions are shown below. (**B**) Average number of Purkinje cells per section for mice at indicated time points; SEM indicated. (**C** and **D**) Cleaved caspase 3 (CC3) immunostaining and merged CC3 and immunofluorescence with DAPI counterstain in the cerebellum of 5-week-old *nm3888*$^{-/-}$ (C) and WT (D) mice. Scale bars, 500 and 50 μm (low magnification and high magnification, respectively) (A) and 20 μm [(C) and (D)]. Multiple *t* tests were performed (B). ****$P \leq 0.0001$, *$P \leq 0.05$.

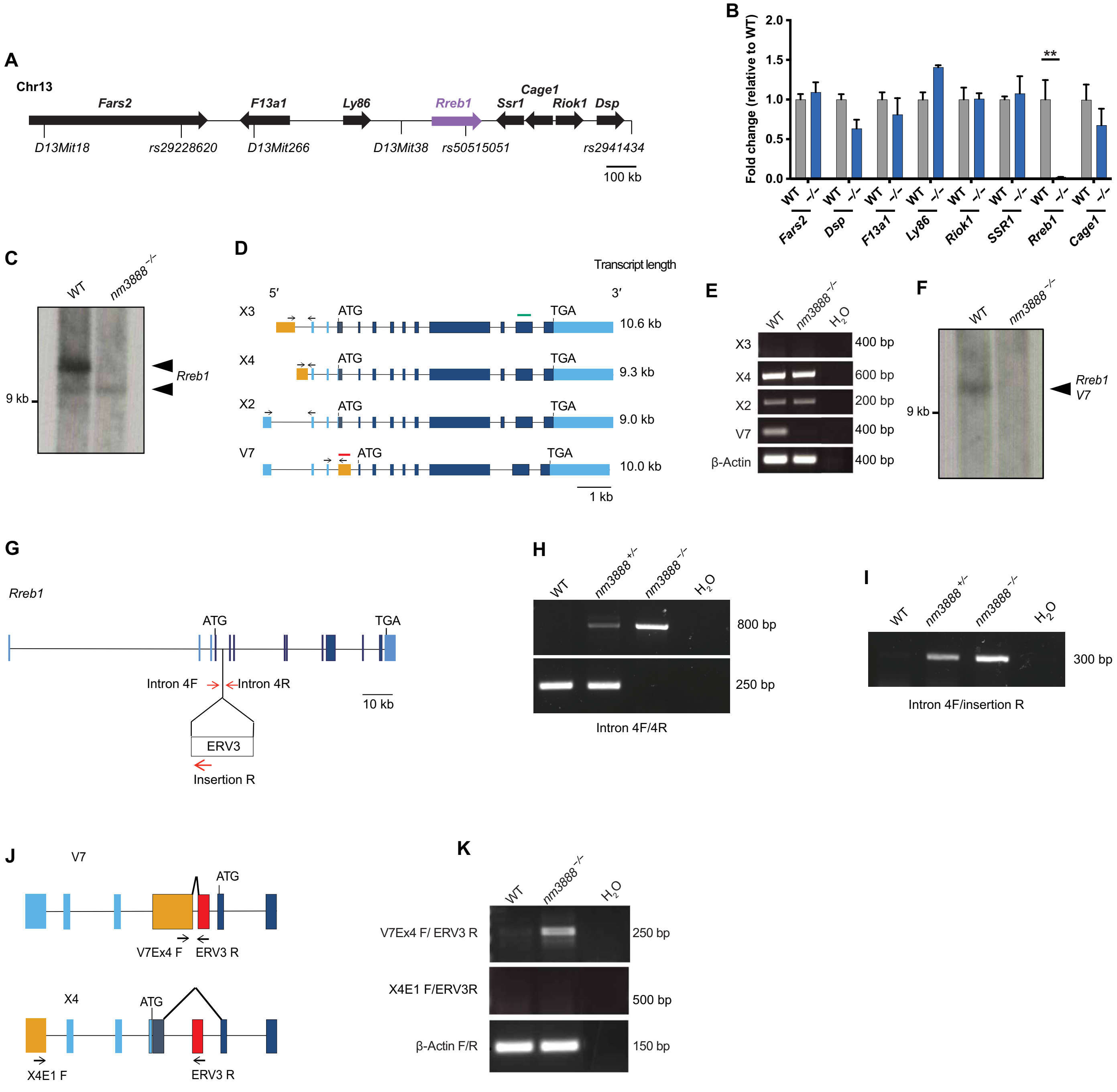


**Fig. 2. The *nm3888* mutation results in loss of *Rreb1*.** (**A**) Schematic of the *nm3888* critical region including protein-coding genes and polymorphisms used for genetic mapping. (**B**) RT-qPCR analysis of protein-coding genes in the critical region from wild-type (WT) and *nm3888*$^{-/-}$ (−/−) cerebella. Mean and SEM are graphed. Only *Rreb1* expression was significantly different between genotypes, $^{**}P \leq 0.001$ (multiple *t* tests with Benjamini and Hochberg correction). (**C**) Northern blot analysis of total *Rreb1* from WT and *nm3888*$^{-/-}$ cerebella. (**D**) Schematics of *Rreb1* transcripts that are 9 kb or greater in length. Coding exons are indicated in dark blue, noncoding exons are in light blue, and transcript-specific exons are in orange. Arrows indicate primers used to amplify transcripts shown in (E). Northern probes used for total *Rreb1* and *Rreb1 V7* are indicated by a green line and red line, respectively. (**E**) RT-PCR of *Rreb1* transcripts shown in (D) from WT and *nm3888*$^{-/-}$ cerebella. (**F**) Northern blot analysis of *Rreb1 V7* from WT and *nm3888*$^{-/-}$ cerebella. (**G**) Schematic of *Rreb1* with coding exons in dark blue and noncoding exons in light blue. Arrows indicate primers used to amplify around and within the *nm3888* insertion. Introns not drawn to scale. (**H** and **I**) PCR analysis of genomic DNA from WT, *nm3888*$^{+/-}$, and *nm3888*$^{-/-}$ mice with primers in *Rreb1* intron 4 (primer pair Intron 4F/Intron 4R). (**J**) Schematic showing splicing of *Rreb1 V7* and *Rreb1 X4* into *nm3888* insertion (red) along with primers used for RT-PCR (arrows). (**K**) RT-PCR for chimeric transcripts containing *Rreb1* and the *nm3888* insertion, performed with cerebellar cDNA from 3-week-old WT and *nm3888*$^{-/-}$ mice. Primers used are indicated on the left.

genes in the critical region, we performed quantitative reverse transcriptase polymerase chain reaction (RT-qPCR) using wild-type and *nm3888*$^{-/-}$ cerebellar cDNA from 3-week-old mice. While we did not observe significant differences in expression of seven of these genes, the expression of a zinc finger transcription factor, *Rreb1*, was decreased 34-fold in mutant cerebella (Fig. 2B). Northern blot analysis for total *Rreb1* demonstrated a loss of the predominant transcript (>9 kb) in *nm3888*$^{-/-}$ brains (Fig. 2C). We performed RT-PCR with primers specific to the four transcripts of *Rreb1* (National Center for Biotechnology Information, https://ncbi.nlm.nih.gov/gene/68750) that were 9.0 kb or greater: XM_006516750.2 (*X2*), XM_006516752.2 (*X3*), XM_006516753.3 (*X4*), and OP867049 (variant 7, hereafter referred to as *V7*). *X2*, *X3*, and *X4* each have different 5′ untranslated regions (5′UTRs) but contain the same coding sequence that produces a protein with 16 zinc fingers. *V7* has a unique 5′UTR, a later ATG start site, and splices around the eighth coding exon found in *X2*, *X3*, and *X4*, producing a protein with 14 zinc fingers (Fig. 2D). We did not detect *X3* in wild-type cerebella, and *X2* and *X4* were expressed at comparable levels to wild-type in the mutant cerebellum (Fig. 2E). However, expression of *V7* was markedly lower in the *nm3888*$^{-/-}$ cerebellum relative to wild-type cerebellum. Northern blot analysis using a probe specific to *V7* demonstrated that it corresponded to the predominant *Rreb1* transcript in the wild-type cerebellum and confirmed its loss in the mutant cerebellum (Fig. 2F).

We examined *Rreb1* in *nm3888*$^{-/-}$ mice to identify the molecular changes that resulted in decreased expression. We sequenced all exons and identified no mutations. However, upon sequencing *Rreb1* introns, we found a 573-bp insertion of an ERV3 retrotransposon element in intron 4 (Fig. 2, G to I), which cosegregated with the degeneration seen in *nm3888* heterozygotes and homozygotes. Given the specific impact of the *nm3888* mutation on expression of *V7*, we hypothesized that disruption of this transcript was due to aberrant splicing from exon 4, which is uniquely found in the *V7* transcript, into the retrotransposon (Fig. 2J). PCR using cerebellar cDNA from *nm3888*$^{-/-}$ mice and primers corresponding to exon 4 of *V7* and the retrotransposon element generated an appropriately sized amplicon (Fig. 2K). Sequencing confirmed that this amplicon was a *V7* exon 4/retrotransposon fusion product. We were unable to amplify an *X4*/retrotransposon fusion product when we used primers corresponding to the unique exon in *X4*, suggesting that the retrotransposon does not disrupt expression of the X4 transcript. Together, our genetic mapping data, identification of the retrotransposon insertion, and altered expression of *Rreb1* strongly suggest that loss of *Rreb1* is causal for the Purkinje cell degeneration seen in *nm3888* mutant mice.

## Expression of *Rreb1 V7* is nervous system specific and temporally regulated

To evaluate whether additional tissues require *Rreb1 V7*, we performed RT-qPCR to assess expression of *Rreb1 V7* in wild-type tissues throughout the body. Although we broadly detected total *Rreb1* using primer pairs that amplify all transcripts of *Rreb1*, *Rreb1 V7* was highly enriched in the brain, spinal cord, and eye (Fig. 3A). To determine which cell types express *Rreb1* in the brain, we performed in situ hybridization with a 3′UTR probe that was complementary to all *Rreb1* transcripts. We detected *Rreb1* in mitral cells of the olfactory bulb, multiple thalamic nuclei, and the retrosplenial area within the cortex (Fig. 3B). In addition, *Rreb1* was highly expressed in Purkinje cells (Fig. 3C), dopaminergic neurons in the substantia nigra pars compacta (Fig. 3D), granule cells of the dentate gyrus of the hippocampus (Fig. 3E), and deep cerebellar nuclei (Fig. 3F). We did not detect *Rreb1* in the *nm3888*$^{-/-}$ brain (Fig. 3G), confirming that *V7* is the predominant transcript expressed in the brain.

To determine whether the temporal expression of the *V7* transcript correlates with the onset of Purkinje cell degeneration, we performed RT-qPCR on wild-type embryos. Although total *Rreb1* was expressed in the developing brain of embryonic day 13.5 (E13.5) embryos, we did not detect *V7* at this time but found that it was expressed at low levels in E18.5 cerebellum (Fig. 3H). Expression levels of both total *Rreb1* and the *V7* transcript were constant in the cerebellum from E18.5 through postnatal day 7 (P7). Between P7 and P14, a period of ongoing cerebellar development, cerebellar levels of *V7* significantly increased. However, the levels of total *Rreb1* did not increase during this time, suggesting that down-regulation of other *Rreb1* isoforms occurs during cerebellar development. Together, these data indicate that both the temporal and spatial expression of the *Rreb1 V7* transcript correlate with the postembryonic and nervous system–specific defects found in *Rreb1 V7*-deficient mice.

## RREB1 regulates the endomembrane system and microtubule network in Purkinje cells

RREB1 directly binds DNA and recruits cofactors that alter histone modifications to potentiate or repress transcription of target genes. Studies of *Rreb1* have generally centered on its role in tumorigenesis, where an increase in copy number and expression of *Rreb1* has been linked to the formation of multiple cancers (*6*–*11*). In addition, total loss of *Rreb1* impairs cell migration during early embryogenesis, resulting in embryonic lethality (*12*, *13*). To determine a role for RREB1 in Purkinje cells, we sought to identify Purkinje cell–specific targets of *Rreb1* via ChIP-seq. To identify targets of *Rreb1 V7*, we generated transgenic mice expressing *V7* with three V5 epitope tags at the C terminus (Fig. 4A). Expression of this transgene was driven by the Purkinje cell–specific promoter, Purkinje cell protein 2 (PCP2) and PCP2 exon 4, which, when placed after the stop codon of a transgene, increases Purkinje cell–specific expression (*14*). Immunofluorescence performed on cerebellar sections from transgenic mice using an antibody to the V5 epitope tag confirmed that the transgene was exclusively expressed in Purkinje cells, and RREB1-V5 localized to the nucleus (fig. S1A).

We performed ChIP-seq in cerebellar lysates from transgenic mice using an antibody against V5. Analysis of these data revealed 3037 peaks, a third of which occurred in introns, another third in intergenic regions, and a third at known promoter regions/transcriptional start sites (Fig. 4B and data S1, ChIP_seq). We assigned 1932 genes to peaks based on proximity, and we identified a strong enrichment of RREB1 binding in protein-coding genes (85%) (Fig. 4C). We performed ChIP-qPCR on three target genes to validate RREB1 binding (fig. S1B). De novo motif analyses demonstrated a significant enrichment ($P = 10^{-925}$) for a Purkinje cell RREB1 binding motif that is highly similar to the binding motif of a known RREB1 cofactor, NEUROD1 (match score = 0.98) (*15*). This de novo motif was enriched in 24% of RREB1 ChIP peaks versus 2.5% of background peaks (fig. S1C). To elucidate cellular processes directly regulated by RREB1, we performed cellular component enrichment analysis on all RREB1 putative target genes. This analysis revealed a significant enrichment for genes associated with the microtubule network, somatodendritic compartment, and the endomembrane system, along with several neurotypical compartments such as the axon and synapse (Fig. 4D and data S1, ChIP_Cell_Comp). Of the top 10 most significantly enriched cellular components, we

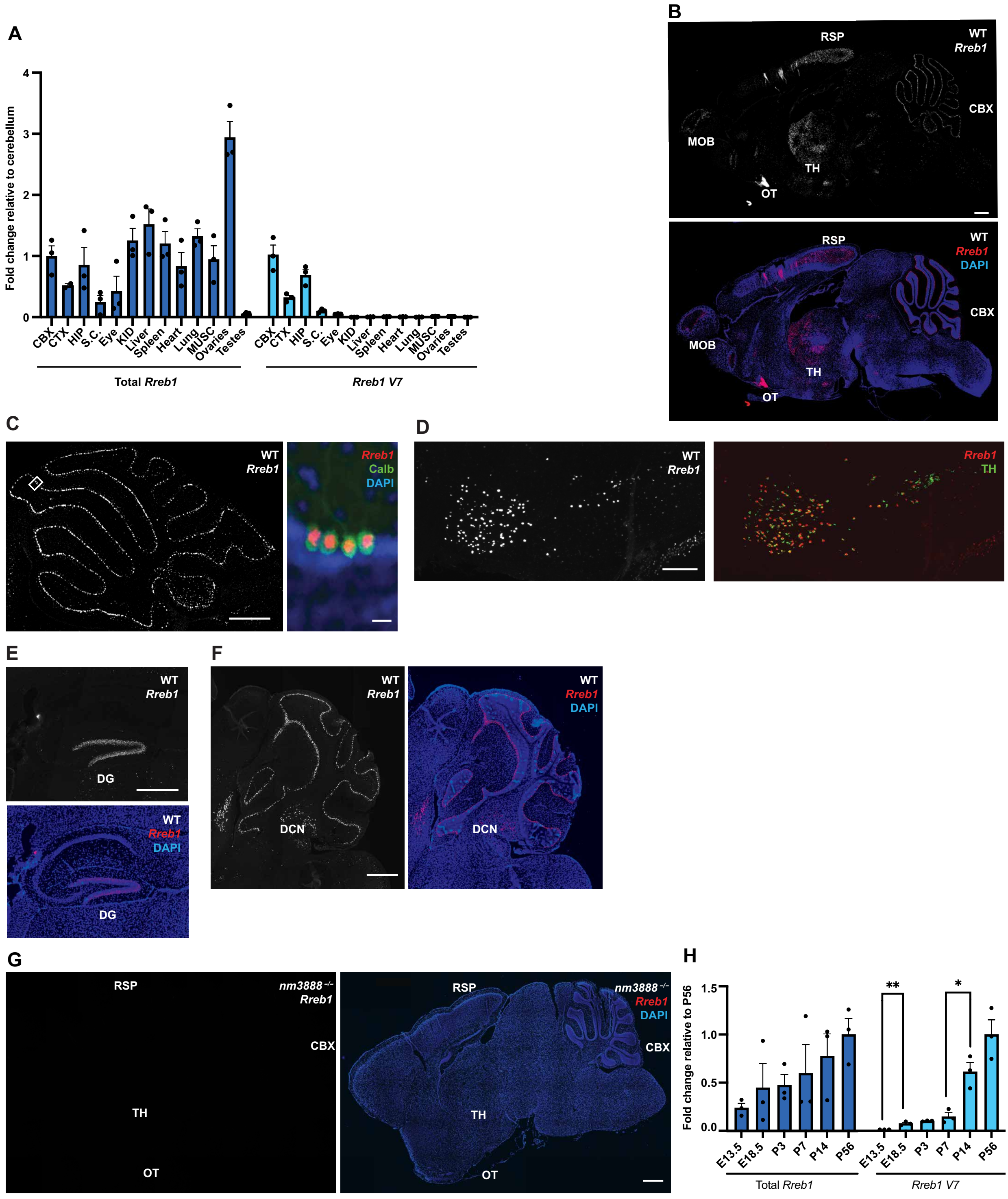


**Fig. 3. *Rreb1 variant 7* (*V7*) is a spatially and temporally regulated transcript.** (**A**) RT-qPCR for total *Rreb1* and *Rreb1 V7* of 3 separate brain regions and 11 tissues from P56 wild-type (WT) mice. Expression is normalized to mean cerebellar (CBX) expression; mean and SEM are graphed. Abbreviations: cerebellum (CBX), cortex (CTX), hippocampus (HIP), spinal cord (S.C.), kidney (KID), and muscle (MUSC). (**B**) *Rreb1* in situ hybridization alone and with DAPI counterstain of WT brain. Abbreviations: olfactory bulb (MOB), olfactory tubercle (OT), thalamus (TH), retrosplenial area (RSP), and cerebellum (CBX). (**C**) Low magnification: *Rreb1* in situ hybridization of WT cerebellum; high magnification: *Rreb1* in situ hybridization with coimmunofluorescence for calbindin D-28 (Calb) and DAPI counterstain of WT cerebellum. (**D**) *Rreb1* in situ hybridization with coimmunofluorescence of tyrosine hydroxylase (TH) of the substantia nigra of WT brain. (**E**) *Rreb1* in situ hybridization and DAPI counterstain of dentate gyrus (DG) of WT brain. (**F**) *Rreb1* in situ hybridization and DAPI counterstain of deep cerebellar nuclei (DCN) of WT brain. (**G**) *Rreb1* in situ hybridization alone, and with DAPI counterstain, of *nm3888*$^{-/-}$ brain. (**H**) RT-qPCR for total *Rreb1* and *Rreb1 V7* at pre- and postnatal time points of WT brain (E13.5) and cerebellum (E18.5 to P56). Average expression relative to mean expression at P56 is graphed with SEM indicated by error bars. Multiple *t* tests were used. $^{**}P \leq 0.01$, $^{*}P \leq 0.05$. Scale bars, 500 μm [(B), (C) (low magnification), (D), (F), and (G)], 10 μm [(C), high magnification], and 200 μm (E). All in situ hybridization was performed on brains from P21 mice.

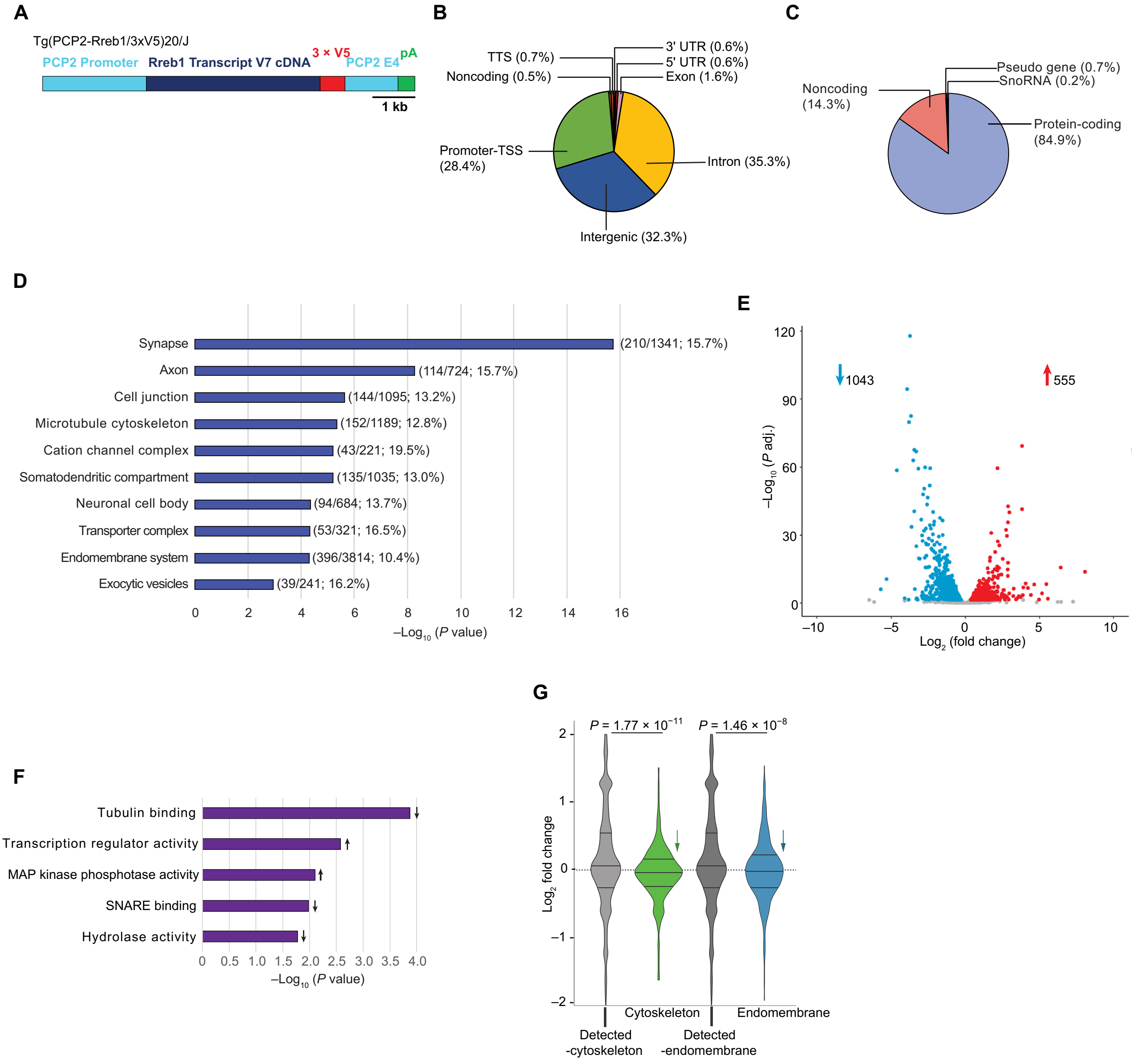


**Fig. 4. RREB1 regulates genes associated with the endomembrane system and cytoskeletal network.** (**A**) Schematic representation of *Rreb1 variant 7* (*V7*) cDNA transgene: PCP2 promoter and fourth exon (light blue), *Rreb1 V7* cDNA (dark blue), three V5 epitope tags (red), and polyA signal (green). (**B**) Breakdown of types of genomic regions containing peaks defined by MACS3 and annotated by HOMER. Transcriptional termination sequence (TTS). (**C**) Breakdown of types of genes associated with MACS3 peaks; gene types annotated by HOMER. (**D**) Cellular component enrichment of putative ChIP targets performed using ShinyGO, plotted by FDR with fraction of ChIP targets/number of genes in category, and percent enrichment [(number of ChIP targets/number of genes in category) × 100%] indicated. (**E**) Volcano plot of gene expression in *nm3888*$^{-/-}$ Purkinje cells relative to wild-type neurons. Up-regulated genes are in red, down-regulated genes are in blue, and genes with a *P* adj ≥ 0.05 are in gray. Numbers of genes up-regulated and down-regulated are indicated. (**F**) Molecular pathway enrichment for significantly differentially expressed genes performed with ShinyGO Molecular Pathway Enrichment; arrows indicate directionality of pathway. (**G**) Violin plot showing differential expression of all cytoskeleton system putative targets (green) compared to all other detected genes (light gray) and differential expression of all endomembrane system putative targets (blue) compared to all other detected genes (dark gray). We determined *P* values by Wilcoxon rank sum tests.

found the largest number of RREB1 putative targets (396) belonging to the endomembrane system, which encompassed other cellular components, including the synapse and exocytic vesicles.

To determine direct and indirect downstream targets of RREB1 in Purkinje cells, we performed RNA-seq from wild-type and *nm3888*$^{-/-}$ Purkinje cells using RiboTag mice (*16*). The hemagglutinin (HA)–tagged ribosomal protein RPL22 was expressed in Purkinje cells using PCP2- CRE, and we immunoprecipitated epitope-tagged ribosomes from *Rpl22*$^{fl/+}$: *PCP2-Cre* mice either homozygous for the *nm3888* mutation or wild type at this locus. We enriched nascent transcripts for polyA$^{+}$ RNA and generated cDNA libraries for sequencing. RNA-seq analysis revealed 1598 differentially expressed genes with approximately

one-third of these genes up-regulated and two-thirds down-regulated in *nm3888*$^{-/-}$ Purkinje cells (Fig. 4E and data S1, RNA_seq_DE). We performed RT-qPCR on three differentially expressed genes to validate expression changes in the *nm3888* mutant (fig. S1D). Molecular pathway analysis of differentially expressed genes revealed a strong enrichment of genes that encode tubulin-binding proteins as well as transcription regulator activity, mitogen-activated protein kinase phosphatase activity, soluble *N*-ethylmaleimide–sensitive factor attachment protein receptor binding, and hydrolase activity (Fig. 4F and data S1, GO_Mol_Path_Upreg and GO_Mol_Path_Downreg). We identified 170 of the differentially expressed genes as RREB1 targets by our ChIP-seq data (fig. S1E and data S1, ChIPseq_RNAseq_Overlap).

We next looked at expression of all putative targets (identified by ChIP-seq) belonging to the endomembrane system and microtubule cytoskeletal network, as both pathways were enriched in our ChIP- and RNA-seq analyses. This analysis revealed that these genes were largely down-regulated in *nm3888*$^{-/-}$ Purkinje cells relative to wild-type cells (Fig. 4G), suggesting that RREB1 plays a role in positively regulating genes associated with both systems.

## Loss of RREB1 alters the microtubule network and dendritic branching

Tubulin-binding proteins govern microtubule dynamics through acetylation, polyglutamylation, tyrosination, and removal of these groups. Loss of tubulin-binding proteins has been associated with alterations in posttranslational modifications of tubulin and subsequent neurodegeneration (*17*). To determine whether the dysregulation of genes encoding tubulin-binding proteins in *nm3888*$^{-/-}$ Purkinje cells led to alterations in posttranslational modification of tubulin and tubulin networks, we performed immunofluorescence with antibodies to tubulin and modified tubulin. We observed a reduction in the intensity of immunofluorescence in *nm3888*$^{-/-}$ Purkinje cells relative to wild-type cells using antibodies to beta III tubulin, acetylated alpha tubulin, and polyglutamylated alpha tubulin in the anterior or mid region of the cerebellum (lobules II and IV) (Fig. 5, A to F). We did not observe a change in Purkinje soma immunofluorescence intensity between genotypes for alpha tubulin and tyrosinated alpha tubulin in this region (fig. S2, A to D). In caudal regions of the cerebellum (lobule IX), we also saw a decrease in beta III tubulin (fig. S2, E and F). However, in contrast to the rostral region of the cerebellum, we saw increased immunofluorescence intensity for alpha tubulin in caudal cerebellum of *nm3888*$^{-/-}$ mice (fig. S2, G and H). Additional differences between the caudal cerebellum of wild-type and *nm3888*$^{-/-}$ mice include an increase in acetylated alpha tubulin (fig. S2, I and J), no altered fluorescence intensity for polyglutamylated alpha tubulin (fig. S2, K and L), and an increase in tyrosinated alpha tubulin (fig. S2, M and N). These data suggest that there are likely different pathways regulating the microtubule network between rostral and caudal regions of the cerebellum.

Immunofluorescence for tyrosinated alpha tubulin in lobule IV revealed alterations in dendritic branching of *nm3888*$^{-/-}$ Purkinje cells. Dendritic arborization of neurons is known to be highly regulated by microtubule-associated proteins, loss of which results in a reduction of dendritic complexity and disorganized arborization (*18*, *19*). To further investigate branching of Purkinje cell dendrites in this region of the cerebellum, we performed cell labeling by injecting lobule IV Purkinje cell soma with biocytin and visualized this with Alexa Fluor 555–streptavidin (Fig. 5, G and I). We tranced dendritic branches from z-stacks of confocal images of Purkinje cells and performed Sholl analyses on the traces (Fig. 5, H and J). We observed a clear reduction in both the branching complexity and the total number of branch points in the dendritic arbors of *nm3888*$^{-/-}$ Purkinje cells (Fig. 5, K and L).

## Loss of RREB1 is associated with alterations in the autophagic system and proteostasis

RNA-seq analysis showed that 59 of 396 RREB1 endomembrane system targets identified by ChIP-seq were significantly differentially expressed in *nm3888*$^{-/-}$ Purkinje cells. Analysis of these genes based on molecular function revealed that 25% of these genes have roles in autophagy (data S1, Endo_Targets).

Autophagy is integral to neuron homeostasis, and loss of genes essential for autophagy has been shown to result in the accumulation of protein inclusions, a hallmark of many neurodegenerative diseases. Given this and our sequencing data, we examined whether the cellular machinery essential for autophagy was compromised in *nm3888*$^{-/-}$ Purkinje cells at 3 weeks of age, before the onset of degeneration. We first examined autophagosomes that engulf cargo bound by the degradation signaling proteins P62 and ubiquitin. As autophagosomes form, microtubule-associated protein 1A/1B-light chain 3 (LC3), a lipidated protein that integrates into the autophagosome membrane, can be visualized as puncta (*20*). To visualize autophagosomes, we crossed *nm3888*$^{-/-}$ mice to mice containing an LC3 transgene conjugated to fluorophores (*CAG-RFP-EGFP-LC3*$^{Tg}$). We counted LC3-positive puncta in Purkinje cells of *nm3888*$^{-/-}$*; CAG-RFP-EGFP-LC3*$^{Tg}$ and *CAG-RFP-EGFP-LC3*$^{Tg}$ mice, and we observed a reduction in the number of LC3 puncta in *nm3888*$^{-/-}$ Purkinje cells (Fig. 6, A and C), suggesting a reduction in the number of autophagosomes.

Autophagosomes fuse with lysosomes forming autolysosomes in which the sequestered cell material is degraded. To determine whether *Rreb1* mutants show altered lysosome numbers in Purkinje cells, we performed immunofluorescence on 3-week-old wild-type and *nm3888*$^{-/-}$ cerebella for lysosomal-associated membrane protein 1 (lamp1), a glycoprotein localized to both late endosomes and lysosomes, and cathepsin-D, a protease localized exclusively to lysosomes (*21*). The number of puncta for both proteins was reduced in *nm3888*$^{-/-}$ Purkinje cells, indicating that, like autophagosomes, fewer lysosomes exist in these mutant Purkinje cells (Fig. 6, A to C). To determine whether loss of *Rreb1* affected lysosome and autophagosome numbers in the caudal region of the *nm3888*$^{-/-}$ cerebellum where Purkinje cells do not degenerate, we examined lysosome and autophagosomes in lobule IX of 3-week-old mutant and wild-type cerebellum. We observed a significant reduction in the number of lamp1 puncta; however, there was no reduction in LC3 or cathepsin-D puncta in lobule IX of the *nm3888*$^{-/-}$ cerebellum (Fig. 6, B and D).

Proteostatic disruption in Purkinje cells has been shown to result in the accumulation of protein inclusions (*22*–*24*). Such inclusions that have failed to be degraded by autophagy or the proteasome contain an accumulation of degradation signaling proteins. These include ubiquitin, which labels proteins for degradation by both autophagy and the proteasome, and ubiquitin-binding protein (P62), an autophagy-specific signaling protein. To identify whether *nm3888*$^{-/-}$ Purkinje cells develop protein inclusions, we performed immunofluorescence for P62 and ubiquitin. We observed P62-positive and ubiquitin-positive puncta and puncta positive for both proteins in *nm3888*$^{-/-}$ Purkinje cells from mice that were 3 to 6 weeks of age (Fig. 6, E to H). Neither *nm3888*$^{-/-}$ nor wild-type Purkinje cells from lobule IX displayed P62-positive or ubiquitin-positive puncta between 3 and 6 weeks of age. Together, these findings suggest heterogeneity in the regulation of proteostasis among Purkinje cells.

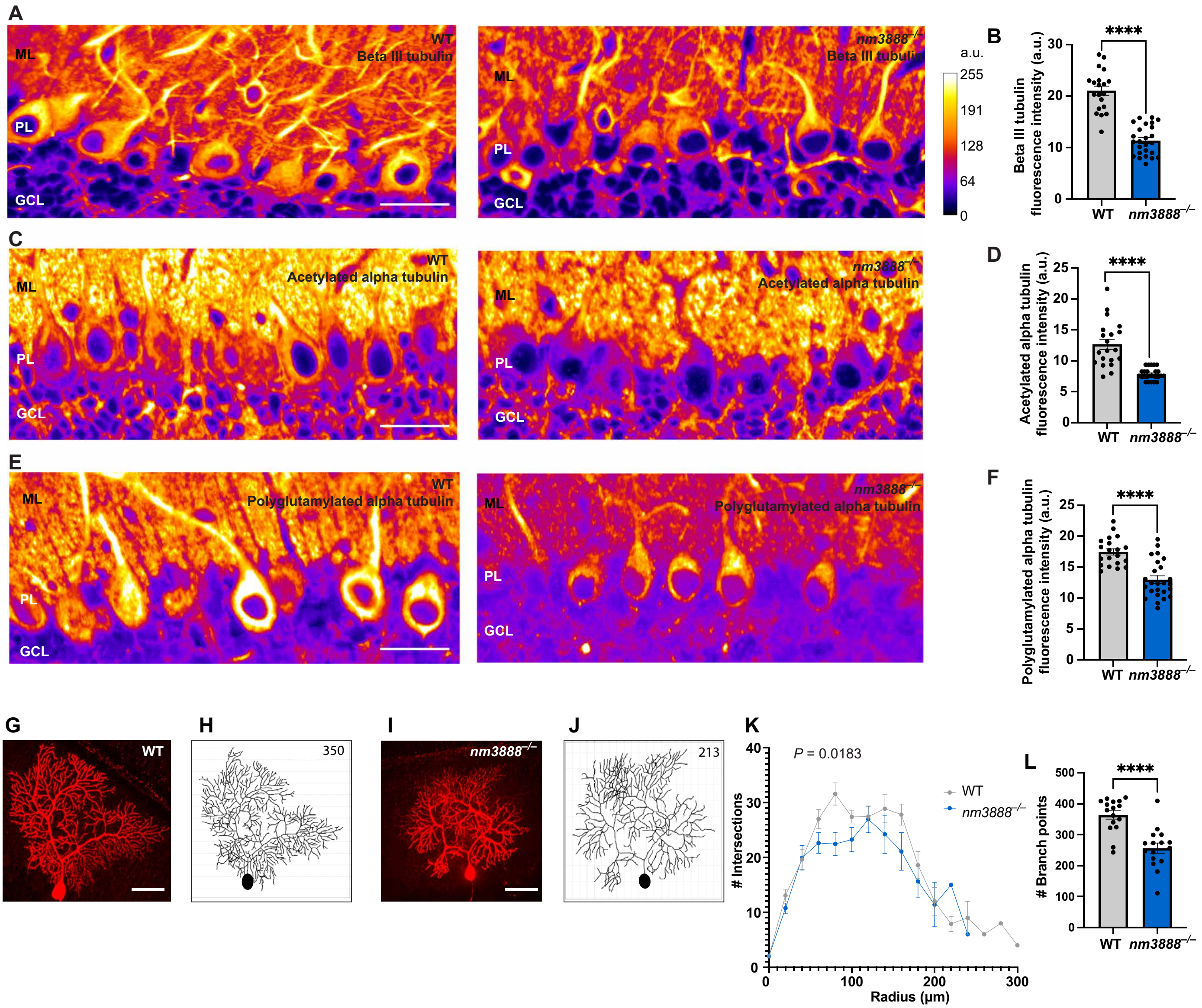


**Fig. 5. Loss of *Rreb1* affects the tubulin network and reduces dendritic branching of Purkinje cells.** (**A**) Immunofluorescence for beta III tubulin in lobule IV and (**B**) average cell soma fluorescence intensity (a.u., arbitrary units) with SEM represented by error bars. (**C**) Immunofluorescence for acetylated alpha tubulin in lobule II and (**D**) average cell soma fluorescence intensity with SEM represented by error bars. (**E**) Immunofluorescence for polyglutamylated alpha tubulin in lobule II and (**F**) average cell soma fluorescence intensity with SEM represented by error bars. [(A), (C), and (E)] Molecular layer (ML), Purkinje cell layer (PL), and granule cell layer (GL) are indicated. Images are pseudocolored on the basis of intensity; an 8-bit LUT scale ranging from 0 to 255 was used. [(B), (D), and (F)] Multiple *t* tests were performed $^{****}P \leq 0.0001$. [(A) to (F)] We used 3-week-old mice for images and quantification. (**G**) Maximal intensity projection and (**H**) dendritic branch tracing of biocytin-labeled Purkinje cells from a wild-type (WT) Purkinje cell. (**I**) Maximal intensity projection and (**J**) dendritic branch tracing of biocytin-labeled dendrites from an *nm3888*$^{-/-}$ Purkinje cell. [(H) and (J)] Total branch points for each cell are indicated in the top right corner of tracing. (**K**) Sholl analysis for wild-type and *nm3888*$^{-/-}$ Purkinje cells from lobule IV in 3-week-old mice. A two-way analysis of variance (ANOVA) was performed, which showed a significant effect for genotype; *P* value is indicated on the graph. (**L**) Graph of average number of branch points for traced dendrites from wild-type and *nm3888*$^{-/-}$ Purkinje cells; SEM is indicated. $^{****}P \leq 0.0001$ (unpaired *t* test). Scale bars, 25 μm [(A), (C), and (E)] and 50 μm [(G) and (I)].

## DISCUSSION

We have demonstrated that loss of *V7*, an *Rreb1* transcript highly expressed in the nervous system, is associated with Purkinje cell degeneration in mice. In addition, we have identified Purkinje cell targets of the zinc finger transcription factor RREB1 and shown that targets of the microtubule network and endomembrane system are positively regulated by RREB1. Loss of *Rreb1* affects proteins associated with the microtubule network, lowering levels of beta III tubulin, acetylated alpha tubulin, and polyglutamylated alpha tubulin. Within the endomembrane system, we observe reduced numbers of autophagosomes and lysosomes as well as the formation of protein inclusions, and apoptosis. Many studies have shown a role for microtubules in autophagy, particularly during the formation of autophagosomes, where microtubule-associated proteins, such as the RREB1 target

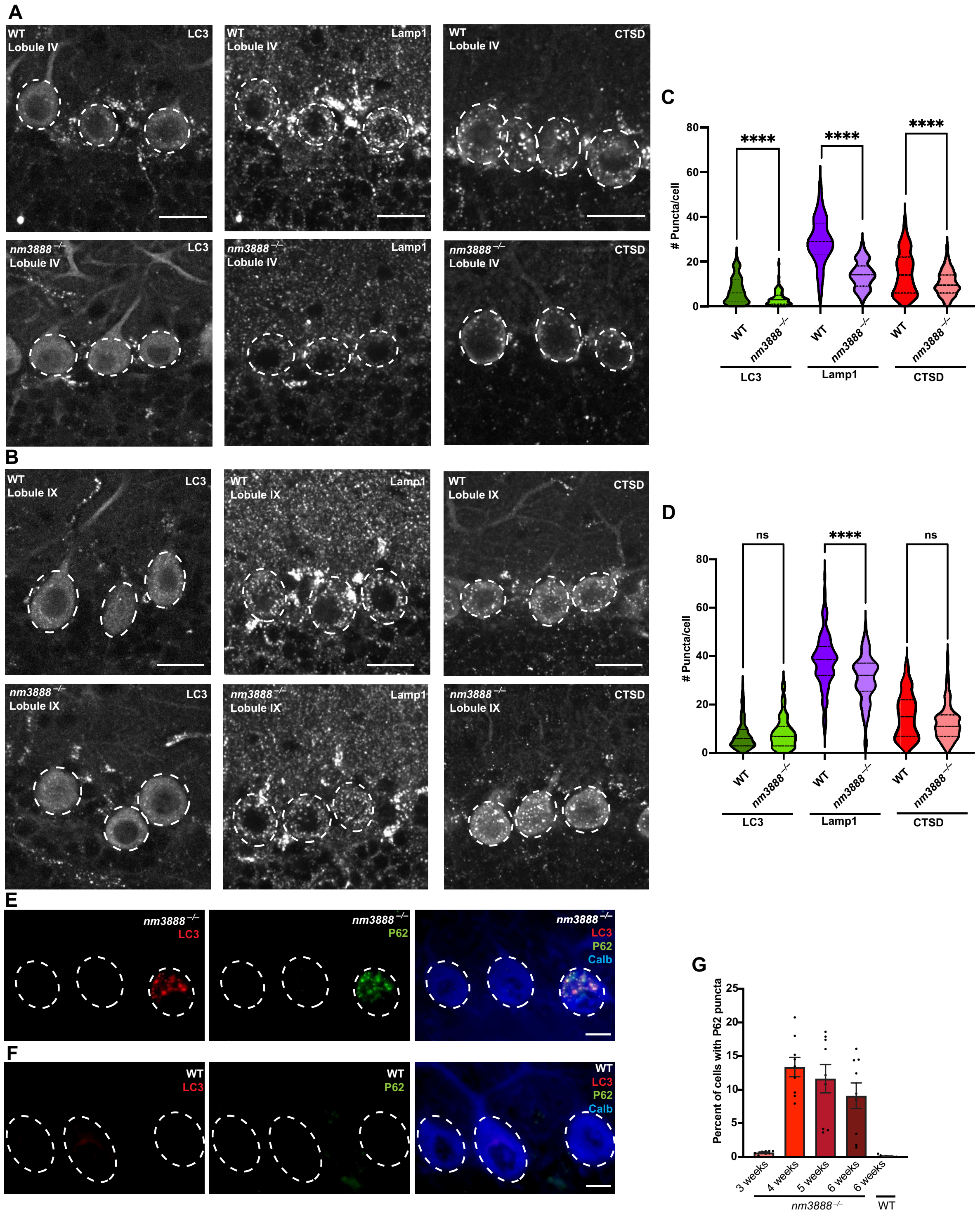


**Fig. 6. Loss of *Rreb1* reduces autophagosome and lysosome abundance and impairs proteostasis.** (**A** and **B**) LC3 (RFP-LC3 fluorescence) and immunofluorescence for lamp1 and cathepsin-D (CTSD) in lobules IV (A) and IX (B) in the cerebellum of 3-week-old CAG-RFP-EGFP-LC3Tg and *nm3888*$^{-/-}$; *CAG-RFP-EGFP-LC3*$^{Tg}$ mice. Purkinje cell soma are encircled in dashed white lines. (**C** and **D**) Puncta per soma of LC3, lamp1, and cathepsin-D (CTSD) in lobule IV (C) and lobule IX (D). Multiple unpaired *t* tests were performed $****P \leq 0.0001$. (**E** and **F**) Immunofluorescence with antibodies to ubiquitin (Ubi), P62, and calbindin D-28 (Calb) with DAPI in the cerebellum of 4-week-old *nm3888*$^{-/-}$ (E) and wild-type (F) mice. (**G**) Percent of Purkinje cells with P62 puncta per section in 3-, 4-, 5-, and 6-week-old *nm3888*$^{-/-}$ mice, and in 6-week-old wild-type (WT) mice. SEM is indicated by error bars. Scale bars, 20 μm [(A) and (B)] and 10 μm [(E) and (F)].

microtubule associated protein 1A (MAP1A), associate with the autophagic membrane component LC3 to facilitate the interaction between LC3 and microtubules (*25*). The maturation of lysosomes is also highly dependent on the microtubule network, and previous studies have demonstrated that inhibition of microtubule dynamics impairs trafficking of proteases essential for protein degradation, such as cathepsin-D, to lysosomes (*26*). Microtubules have also been shown to have an essential role in the formation of autolysosomes where depolymerization of the microtubule network impedes colocalization of autophagosomes and lysosomes at the microtubule organizing center (*27–30*). These studies suggest that disruption of the microtubule network may affect proteostasis in *Rreb1*-deficient Purkinje cells.

The microtubule cytoskeleton also plays an integral role in nervous system development (*31–33*). Studies have also demonstrated that tubulin and microtubule-binding proteins that regulate the microtubule cytoskeleton are critical for neuron migration during development (*34*, *35*) and dendritic arborization (*36*, *37*), where loss of these proteins results in improper or underdeveloped brain structures and altered dendritic complexity. Among the 23 differentially expressed somatodendritic targets of RREB1, two belonged to the stathmin family of tubulin-binding proteins [Stathmin2 (STMN2) and STMN4], which have been shown to promote microtubule catastrophe and inhibit polymerization, thereby influencing microtubule dynamics (*38*, *39*). Previous studies have demonstrated roles for stathmins during Purkinje cell dendritic arborization, where altered activity of these stathmins results in decreased dendritic branching, which is rescued with transgenic expression of the missing stathmin protein (*40*, *41*). Thus, loss of *Rreb1* may directly impair Purkinje cell dendritic development through dysregulation of STMN2 and STMN4. In addition, STMN2 loss has been shown to drive axon degeneration, motor neuropathy, and neuromuscular junction denervation and has been implicated in amyotrophic lateral sclerosis pathogenesis (*42–44*). It is unknown whether reduced levels of STMN2 contribute to Purkinje cell degeneration in *nm3888*$^{-/-}$ mice. Future studies will investigate whether transgenic expression of STMN2 in *Rreb1*-deficient mice can alter the course of Purkinje cell degeneration.

While there are no reported human cases of homozygous loss of *hRREB1*, heterozygous loss of *hRREB1* has been reported in patients with interstitial microdeletions of chromosome 6p (*45*). All patients presented with intellectual disability, craniofacial dysmorphisms, and cardiac abnormalities, consistent with Noonan-spectrum disorder, and many of these features, including cardiac defects and craniofacial abnormalities, have also been observed in mice heterozygous for *Rreb1* loss. These deficits, as well as the early embryogenesis defects in *Rreb1* knockouts (*12*, *13*), underscore the multiple roles of *hRREB1* in development. In contrast, *nm3888* mutant mice have a hypomorphic reduction in *Rreb*1 with loss of a nervous system–specific transcript, *V7*, resulting in postnatal phenotypes rather than early developmental defects. Mice homozygous and heterozygous for the *nm3888* mutation provide models of neurological phenotypes associated with loss of *Rreb1* that may prove fruitful in ongoing efforts to understand postnatal phenotypes incurred by patients experiencing heterozygous loss of *hRREB1*.

Our Northern blot analysis of total *Rreb1* in whole mouse brain followed by RT-qPCR suggests the expression of two protein products in the brain. The *V7* transcript encodes one protein isoform with a length of 1618 amino acids and 14 zinc fingers, while the remaining transcripts expressed in the brain encode a protein isoform with a length of 1754 amino acids and 16 zinc fingers. The differences in protein structure occur in the N terminus where the V7 isoform contains a later, in-frame ATG start site, resulting in one fewer zinc finger at the N terminus, and in lack of exon 12, which contains another zinc finger. Because these sequences are nearly identical (95%), it is possible that the functional differences in mouse RREB1 and human *hRREB1* protein isoforms are predominantly dictated by their unique spatiotemporal expression patterns, and thus cellular context, rather than by structural differences. Alternatively, given the ability of zinc fingers to bind DNA and proteins, it is possible that the zinc finger difference at the N terminus and in exon 12 alters DNA binding, resulting in unique targets for each protein isoform, or in unique interactions with binding partners when recruiting complexes or cofactors to regulate gene expression.

Our study has identified gene targets of RREB1 in Purkinje cells that have previously been associated with the endomembrane system and the regulation of autophagy. In addition, *Rreb1*-deficient Purkinje cells have reduced numbers of autophagosomes and lysosomes and develop protein inclusions. Future studies are necessary to establish a direct connection between dysregulation of RREB1 target genes, alterations in autophagic and lysosomal machinery, the formation of protein inclusions, and Purkinje cell degeneration.

## MATERIALS AND METHODS

### Mice

All mouse studies were under the guidance of and approved by the Institutional Animal Care and Use Committees of the University of California, San Diego and the Jackson Laboratory. We housed mice in a 12-hour light/dark cycle with free access to food and water. Where possible, we compared littermates of wild-type and *nm3888*$^{-/-}$ mice within each experiment. We used mice from both sexes for all experiments. We obtained B6N.129-Rpl22tm1.1Psam/J (stock no. 011029), *C57BL/6-Tg*(*CAG-RFP/EGFP/Map1lc3b*)*1Hill/J* (stock no. 027139), and *B6.129-Tg*(*Pcp2-cre*)*2Mpin/J* (stock no. 004146) mice from the Jackson Laboratory. These mice have been previously described (*16*, *46*).

The *nm3888* mutation arose spontaneously on the NOD/ShiLtJ (NOD) background. We genotyped mice using the following primers: wild-type allele forward: 5′ATGCCTGTCTGTCCATCTCT3′; reverse: 5′TCCAGAGGCAGAAGGAATGA3′; mutant allele forward: 5′AGCCTGAGCACAGAAGGAAC3′; reverse: 5′GACATTGGACGGTTGGAGAT3′. For genetic mapping of the *nm3888* mutation, we intercrossed NOD.*Rreb*$^{nm3888/+}$ mice with CAST/Ei mice and the resulting F1 *Rreb1 nm3888*$^{+/-}$ mice to generate F2 *Rreb1nm3888*$^{-/-}$ mice ($n$ = 402). We assessed ataxia and cerebellar histology of F2 *Rreb1*$^{nm3888-/-}$ mice at 6 weeks of age. We performed genome scans on F2 mice using polymorphic microsatellite markers and fine mapping using these, and additional, single-nucleotide polymorphism markers. Because of difficulties in aging NOD-*Rreb*$^{nm3888/+}$ mice, which often develop diabetes by 3 months of age, we crossed the *nm3888* allele onto the C57BL6/J background (*B6J.nm3888*). To generate congenic *B6J.Rreb1*$^{nm3888}$ mice, we intercrossed NOD-*Rreb*$^{nm3888/+}$ and B6J mice, and we backcrossed mice carrying the *nm3888* mutation to B6J mice for seven generations. *B6J.nm3888*$^{-/-}$ mice displayed the same onset of degeneration as NOD *nm3888*$^{-/-}$ mice.

To generate transgenic *Tg*(*PCP2-Rreb1/3xV5*)*20/J* mice, we subcloned the coding sequence of *Rreb1* transcript *V7* cDNA with triple C-terminal V5 tags inserted before the stop codon into the BamHI site of the fourth exon of the L7/PCP2 minigene (*14*). We linearized the construct and injected this into the pronucleus of C57BL/6J zygotes. We

identified founders by PCR using primers that recognize tandem repeats of the transgene (forward: 5′TCTGACCAAATACCACCACC3′; reverse: 5′CAGATACTGCATGCGTGGTC3′).

### Immunohistochemistry
For immunofluorescence with antibodies to alpha tubulin, beta III tubulin, acetylated alpha tubulin, polyglutamylated alpha tubulin, and tyrosinated alpha tubulin, we used four wild-type mice and five *nm3888*$^{-/-}$ mice. We performed all other immunohistochemistry and immunofluorescence experiments on three wild-type mice and three mice with the *nm3888* mutation. We transcardially perfused anesthetized mice with acetic acid/methanol for immunohistochemistry or immunofluorescence of calbindin-D28 (mouse, Sigma-Aldrich, C9848, 1:1000 or rabbit, Swant, cb38, 1:1000), P62 (guinea pig, American Research Products, 03-GP62-C, 1:300), or ubiquitin (mouse, Cell Signaling Technology, 3936, 1:350); 10% neutral-buffered formalin for beta III tubulin (mouse, Abcam, ab78078, 1:1000), alpha tubulin (mouse, Sigma-Aldrich, T6074, 1:1000), polyglutamylated alpha tubulin (mouse, Sigma-Aldrich, T9822, 1:500), tyrosinated alpha tubulin (mouse, Sigma-Aldrich, T9028, 1:500), and acetylated alpha tubulin (mouse, Sigma-Aldrich, T6793, 1:400); or 4% paraformaldehyde for V5 (mouse, Thermo Fisher Scientific, R960-25, 1:700), lamp1 (rat, University of Iowa, 1D4B, 1:500), and cathepsin-D (rat, R&D Systems, MAB1029, 1:300). We postfixed tissues overnight and subsequently embedded them in paraffin; for cryosections, we incubated samples in 30% sucrose in phosphate-buffered saline (PBS) for 2 days before embedding them in Tissue Freezing Media (Electron Microscopy Sciences, no. 72592-C). For immunohistochemistry, we deparaffinized 7-μm paraffin sections and rehydrated them before antibody staining. After we incubated sections with primary antibodies, we incubated them with goat anti-mouse:Biotin (Sigma-Aldrich, B7264, 1:100) and ExtrAvidan (Sigma-Aldrich, E2886, 1:50) before diaminobenzadine (Sigma-Aldrich, D4293-50SET) visualization. We counterstained slides with hematoxylin before cover slipping. For immunofluorescence, we performed antigen retrieval on sections fixed in 4% paraformaldehyde or 10% neutral-buffered formalin by microwaving deparaffinized slides at low power for three cycles of 3 min in 0.01 M sodium citrate buffer (pH 6.0, 0.05% Tween-20) separated by 3-min intervals of rest. We diluted all secondaries (goat anti-rabbit, AF488, Invitrogen, A11008; goat anti-mouse, AF488, Invitrogen, A11029; goat anti-rat:AF555, Invitrogen, A21434; donkey anti-rat, AF488, Invitrogen, A21208; goat anti-rabbit, AF305, Invitrogen, A21068; goat anti-guinea pig, AF488, Invitrogen, A11073; goat anti-mouse, AF555, Invitrogen, A21424) at 1:500 in 5% goat serum (Jackson ImmunoResearch, 005-000-121) and PBS-T (0.3% Triton X-100). We stained nuclei with 4′,6-diamidino-2-phenylindole (DAPI) and quenched autofluorescence with Sudan Black.

### Biocytin labeling
We anesthetized 3-week-old mice with ketamine/xylene mixture and perfused them with ice-cold sucrose cutting solution (210 mM sucrose, 3 mM KCl, 26 mM $NaHCO_3$, 1.25 mM $NaH_2PO_4$, 10 mM glucose, $MgSO_4$, 0.5 mM $CaCl_2$, and 3 mM sodium ascorbate, pH 7.3 to 7.4, 300 to 310 mosmol/kg). We prepared six 300-μm-thick sagittal cerebellum sections using a vibratome (VT1200S, Leica) in ice-cold sucrose cutting solution. We recovered the slices in oxygenated artificial cerebrospinal fluid at 30°C for 30 min and then kept them at room temperature (25°C) at least an hour before use. In each section, we injected one Purkinje neuron in lobule IV with biocytin (2 mg/ml) in internal solution containing 145 mM K-gluconate, 5 mM NaCl, 10 mM Hepes, 1 mM $MgCl_2$, 0.2 mM EGTA, 2 mM MgATP, and 0.1 mM $Na_3GTP$ [(pH 7.3) with KOH, 293 mOsm]. We next transferred the section to 4% paraformaldehyde for 1-hour incubation at room temperature. We blocked sections for 1 hour in 5% goat serum and then incubated them overnight in streptavidin, AF555 (1:500, Invitrogen, S32355). We washed slices three times for 10 min in PBS-T (1× PBS, 0.03% Triton X-100) before cover slipping with Fluoromount (Southern Biotech, 0100-01). We used three wild-type and three *nm3888*$^{-/-}$ mice.

### In situ hybridization
We used an RNAScope probe for *Rreb1* (ACDBio, 542641-C2) for in situ hybridization on two wild-type and two *nm3888*$^{-/-}$ mice following the manufacturer's protocol. Briefly, we transcardially perfused mice with 10% neutral-buffered formalin and processed and embedded brains for paraffin sectioning. We baked 7-μm sections at 60°C for 1 hour before deparaffinization. We treated sections with hydrogen peroxide (ACDBio, 322281) before antigen retrieval in target retrieval buffer (ACDBio, 322000) and treatment with protease plus (ACDBio, 322281). We incubated sections with the probe for 30 min and then washed them in 1× wash buffer (ACDBio, no. 310091). We stored slides in 5× SSC overnight before a series of amplification steps and incubation with Cy3 (PerkinElmer, FP1170, 1:1500). For immunofluorescence after in situ hybridization, we incubated either calbindin D-28 (rabbit, Swant, cb38, 1:1000) or tyrosine hydroxylase (mouse, Leica, NCL-L-TH, 1:250) on slides overnight, then incubated them with secondary antibody. We stained nuclei with DAPI and slides and stored them in the dark at 4°C before imaging.

### Imaging
We generated images for calbindin D-28 immunohistochemistry using an Olympus VS200 SlideScanner. We took in situ images (tiled) and tubulin and aggregate immunofluorescence images using a Zeiss Axiovert 200. We took images for V5 and calbindin immunofluorescence of *Tg*(*PCP2-Rreb1/3xV5*)*20/J* on a Leica SP5 confocal microscope. We imaged dye-filled neurons and images used for quantification of lamp1, cathepsin-D, and LC3 using a Nikon Ti-2 Eclipse and CSU W1 SORA confocal with a variable sized stack custom to each section and 0.3-μm z stepsize.

### RNA extraction
We euthanized three wild-type and three *nm3888*$^{-/-}$ mice, dissected out organs and flash froze them in liquid nitrogen for storage at −80°C. We homogenized tissues in 1 ml of TRIzol (Life Technologies, 15596018) using a polytron homogenizer and isolated RNA as per the manufacturer's protocol. We determined concentration and A260/A280 of isolated RNA by Nanodrop.

### Reverse transcription and PCR
We performed cDNA synthesis on deoxyribonuclease-treated total RNA from three wild-type and three *nm3888*$^{-/-}$ mice using oligo(dT) primers and the SuperScript III First-Strand Synthesis System (Invitrogen, 18080051). We performed quantitative RT-PCR using SYBR Green Supermix (Bio-Rad, 1708880). We performed each quantitative RT-PCR reaction with 40 ng of cerebellar cDNA. For tissue panel and developmental time course of *Rreb1* expression, we used the following primers: Total *Rreb1*, forward: 5′GTCACATGCTGGTGCACTCT3′, reverse: 5′CGTGACTCAGCTTCCTTTTG3′; *Rreb1 V7*, forward:

5′CTGTGGGTGCCTGAGAAAAT3′, reverse: 5′GGGGACAGTTGTAGGAAGAC3′, ETF1 as a control, forward: 5′GGAACGTGGAGATCTGGAAG3′, reverse: 5′GCCACTCGTGAAATCTGGT3′. For RT-qPCR of genes in the critical region, we used the following primers: *Cage1*, forward: 5′TTCGAGCAGCTTGACTTGA3′, reverse: 5′TGTGACTTCTTCCTGGTGAC3′; *Dsp*, forward: 5′GAGAAATTCCAAAAGCAGGCT3′; reverse: 5′CCTGAACAACTTGAGTGCAC3′; *F13a1*, forward: 5′TGGGGACTTCAAAGACATCAA3′, reverse: 5′AGGTCCATCTCAGCTTTGTC3′; *Fars2*, forward: 5′GCCTTTTCTGGAGTGAGGAT3′; reverse: 5′GTGTAGTTCTCAGAGGGCAG3′; *Ly86*, forward: 5′AGTGTTCCAAGCAGATCCAA3′; reverse: 5′TTGCCATCAGAGTTATGTCCA3′; *Riok1*, forward: 5′AGGAAAATGGTGAGGACGTG3′, reverse: 5′CTGATTGGTTCTGGACATGGT3′; *Ssr1*, forward: 5′CTTCATCCCTGCAGAGCC3′, reverse: 5′CCATCTAACCCGTCCTCTCT3′; *Rreb1*, forward: 5′CTGTGGGTGCCTGAGAAAAT3′, reverse: 5′GGGGACAGTTGTAGGAAGAC3′; *Gapdh* control, forward: 5′CATTGTCATACCAGGAAATG3′, reverse: 5′GGAGAAACCTGCCAAGTATG3′. For analysis, expression levels of each gene measured was normalized to a loading control using the $2^{-\Delta\Delta CT}$ method (*47*). For RT-PCR of chimeric transcripts, we used the following primers: V7-ERV3 primers, forward: 5′CTTCGTGTGCCTCTCTTCCT3′; reverse: 5′GAGCCCACGGTTAAGAGACT3′; X4-ERV3 primers, forward: 5′GGGCATGCATTTGGTCCTAG3′; reverse: 5′GAGCCCACGGTTAAGAGACT3′. For RT-qPCR of *Rreb1* transcripts at and over 9.0 kb, we used the following primers: *X2*, forward: 5′CGCGGTGTACTTCTGAAACT3′; reverse: 5′CCTCAAAGCCACCACTTCTG3′;*X4*,forward:5′GGGCATGCATTTGGTCCTAG3′; reverse: 5′CCTCAAAGCCACCACTTCTG3′; *X3*, forward: 5′GGGCCTGTTTAGCTATGGTG3′; reverse: 5′CCTCAAAGCCACCACTTCTG3′; *V7*, forward: 5′GGACTATCATTTGGGGCAGAA3′; reverse: 5′AGGGTTTTCTCCATGCAAGG3′.

## Northern blot analysis

We pooled total RNA from three wild-type and three *nm3888*$^{-/-}$ mice and selected mRNA by oligo(dT) cellulose, and 5 μg was electrophoresed on a 1% formaldehyde agarose gel. We transferred samples to a nylon membrane (Amersham, RPN303B) and ultraviolet–cross-linked this membrane. We generated *Rreb1* probes to the eighth coding exon using PCR (forward primer, 5″′CACAGACACGTTGACCACC3′; reverse primer, 5′GCACATGAGGTCACACACAG3′) or to the fourth noncoding exon in *V7* (forward primer, 5′AGAGGAAAGCCCTTGACACA3′; reverse primer, 5′CTGTGGGTGCCTGAGAAAAT3′) to detect all transcripts and *V7*, respectively. We radiolabeled probes with α-$^{32}$P deoxycytidine triphosphate and performed hybridization overnight at 42°C. We carried out membrane wash steps at 65°C at increasing concentrations of SDS in 2× SSC buffer. We repeated this experiment twice with two sets of pooled mice.

## RiboTag RNA extraction and library construction

We isolated cerebella from three Rpl22$^{fl/+}$; PCP2-Cre$^{Tg/+}$ and four Rpl22$^{fl/+}$; PCP2-Cre$^{Tg/+}$; *nm3888*$^{-/-}$ mice at P21 and homogenized them using a dounce homogenizer in 1.5 ml of lysis buffer [1% NP-40, 100 mM KCl, 50 mM tris (pH 7.4), 12 mM $MgCl_2$, cycloheximide (0.1 mg/ml), protease inhibitor cocktail (Sigma-Aldrich, 11836170001), heparin (1 mg/ml), and RNAseOUT (5 μl/ml; Thermo Fisher Scientific, 10777019)]. We then isolated ribosomes as previously described (*16*). Briefly, we incubated lysates containing tagged ribosomes for 4 hours at 4°C with 5 μg of rat anti-HA antibody (Roche, 11867423001) after which we added 400 μl of protein G–coupled Dynabeads (Thermo Fisher Scientific, 10004D) to the lysate and incubated these samples at 4°C overnight. The following day, we washed ribosome-bound beads three times with high salt buffer [0.3 M KCl, 1% NP-40, 50 mM tris (pH 7.4), 12 mM $MgCl_2$, cycloheximide (0.1 mg/ml), and 0.5 mM dithiothreitol] and isolated mRNA using buffer RLT and spin columns (Qiagen RNEasy Mini Kit, 74104). We eluted RNA in 30 μl of $H_2O$ and evaluated the quality and quantity using high-sensitivity RNA ScreenTape (Agilent, 5067-5576), an Agilent tapestation analyzer (Agilent, G2992AA), and a Qubit 2.0 fluorometer (Life Technologies). We generated three libraries (using one mouse per library) for each genotype using the Kapa Stranded mRNA-seq Kit (KAPA, KK8400) in accordance with the manufacturer's instructions, and we obtained 100-nt paired-end reads using an Illumina NovaSeq 6000.

## ChIP and ChIP-seq library construction

We used cerebella from two independent biological replicates. We froze cerebella from *B6J.Tg*(*PCP2-V7-3V5*) mice at P21 in liquid nitrogen. We minced tissue in 1% formaldehyde-PBS and incubated samples for 10 min, rotating, at room temperature. We added glycine at a final concentration of 0.125 M to terminate cross-linking and incubated the samples an additional 10 min before washing them three times in PBS. After the final wash, we resuspended tissue in 600 μl of ChIP lysis buffer [50 mM tris-HCl (pH 7.5), 0.150 M NaCl, 1 mM EDTA, 1% Igepal CA-630 (Sigma-Aldrich, I8896), 0.25% sodium deoxycholate, 0.10% SDS, and protease inhibitor cocktail (Roche, 11836170001)], and homogenized them with a plastic pestle and incubated them on ice for 10 min. We performed ChIP-seq as previously described (*48*). Briefly, we sonicated lysates for 10-s on, 30-s off, for 12 cycles at 20% amplitude with 2-min rest on ice, then 10 cycles at 30% amplitude with 2-min rest on ice, followed by 12 cycles at 30% amplitude. We ran 1 μl of each sample on the Tape Station using high sensitivity D1000 screen tape (Agilent, 5067–5584) to test sonication efficacy. We set aside 5-μl aliquots of each sample as input, and we added 50 μl of V5 antibody conjugated to magnetic beads (MBL International, M167-11) to the remaining lysate, which was incubated overnight at 4°C. After three washes with 200 μl of wash buffer I [20 mM tris-HCl (pH 7.5), 150 mM NaCl, 0.10% SDS, 1% Triton X-100, 2 mM EDTA, and 1× protease inhibitors], three washes with 200 μl of wash buffer II [250 mM LiCl, 1% Triton X-100, 0.70% sodium deoxycholate, 10 mM tris-HCl (pH 7.5), 1 mM EDTA, and 1× protease inhibitors], and two washes in 180 μl of TET buffer [10 mM tris-HCl (pH 7.5), 1 mM EDTA, 0.20% Tween 20, and 1× protease inhibitors], we resuspended bead-bound samples in 80 μl of elution buffer [10 mM tris-HCl (pH 8.0), 0.50% SDS, 5 mM EDTA, and 280 mM NaCl] and reverse cross-linked with 1 μl of proteinase K (20 mg/ml) for 1 hour at 55°C followed by 1 hour at 65°C. We performed library preparation for ChIP samples on beads as previously described (*48*) using the NEB DNA Ultra II Kit (NEB, E7645S). We used adapters from KAPA (KAPA, KR1317). We ran libraries on a NovaSeq 6000, which generated 75-nt single-end reads.

## Quantification and statistical analyses

### *Quantification of Purkinje cell numbers*

To quantify Purkinje cell numbers, we used three mice for each time point and genotype. For each mouse, we collected two sections 7 μm thick and a minimum of 50 μm apart, stained for calbindin-D28 (mouse, Sigma-Aldrich, C9848), and counterstained with hematoxylin. We quantified Purkinje cells from each section and independently

plotted these on the bar graph in Fig. 1. Statistical analyses are indicated in the legend for Fig. 1.

### RT-qPCR analysis

For quantitative RT-PCR experiments, we evaluated expression in cerebella from three wild-type mice and three *nm3888*$^{-/-}$ mice. We normalized expression levels of the genes of interest to *Etf1* or *Gapdh* using the $2^{-\Delta\Delta CT}$ method (*47*).

### Quantification of immunofluorescence intensity

We used one section that was 7 μm thick from each of four wild-type and five *nm3888*$^{-/-}$ mice. We deparaffinated and stained sections together. We used five neurons from each section to quantify immunofluorescence intensity. We opened images in ImageJ and drew an ellipse around the cell soma of a given Purkinje cell, measured the integrated density, and plotted this in the bar graph in Fig. 5. Statistical analyses are indicated in the legend for Fig. 5.

### Quantification of dendritic branching

To quantify the number of dendritic branch points in biocytin-labeled Purkinje cells, we used the plugin Simple Neurite Tracer in ImageJ to trace dendritic arbors of each cell in three-dimensional space. We used three wild-type and three *nm3888*$^{-/-}$ mice for this experiment, and we traced between five and six neurons for each animal. Statistical analyses are indicated in the legend for Fig. 5.

### Sholl analysis

We used the Simple Neurite Tracer plugin for ImageJ to perform a Sholl analysis. We used a radius of 20 μm and generated a polynomial trendline using the "Best Fitting Degree" determined by the plugin algorithm. Statistical analysis is indicated in the legend for Fig. 5.

### Quantification of LC3, lamp1, and cathepsin-D puncta

For lamp1, LC3, and cathepsin-D puncta quantification, we analyzed a minimum of 30 Purkinje cells in cerebellar lobule IV and lobule IX per section and analyzed one section from each of three wild-type and three *nm3888*$^{-/-}$ mice. For quantification, we used the ImageJ plugin puncta analyzer with a rolling ball radius set to 50 and minimum puncta size set to four pixels. For each cell, we manually adjusted the threshold for puncta to detect individual puncta. Statistical analyses are indicated in the legend for Fig. 6.

### Quantification of P62 and P62 + ubiquitin-positive puncta

For inclusion quantification, we quantified the total number of P62-positive Purkinje cells (as marked by calbindin D-28 positivity), in total cerebella for three 7-μm sections spaced 100 μm apart per mouse. We performed analyses for three *nm3888*$^{-/-}$ mice at each time point and three 6-week-old wild-type mice. Statistical analyses are indicated in the legend for Fig. 6.

### Analysis of sequencing data

For RNA-seq, we deduplicated reads and trimmed them with fastp (*49*) before alignment to the mm10 genome with Bowtie2 (*50*). We used featurecounts (*51*) to calculate read counts for each gene in the mm10 genome, used DESeq2 (*52*) for differential expression analyses, and used batch as a covariant. We used ShinyGO (*53*) for molecular pathway enrichment and input significantly up-regulated and significantly down-regulated genes separately.

For ChIP-seq, we trimmed reads with fastp (*49*) to remove adapter sequences before alignment to the mm10 genome with Bowtie2 (*50*). We used Sambamba (*54*) to filter out multimappers and deduplicate reads and used MACS3 (*55*) to perform peak calling using the following parameters: -g 1.87e9, -q 0.05. We merged samples using bedtools where only overlapping peaks were merged. For gene ontology enrichment, we used ShinyGO (*53*). We used Homer (*56*) for de novo and known motif enrichment analysis with default parameters.

## Supplementary Materials

**This PDF file includes:**
Figs. S1 and S2
Legend for data S1
Legends for movies S1 and S2
References

**Other Supplementary Material for this manuscript includes the following:**
Data S1
Movies S1 and S2

## REFERENCES AND NOTES


1. P. Sweeney, H. Park, M. Baumann, J. Dunlop, J. Frydman, R. Kopito, A. McCampbell, G. Leblanc, A. Venkateswaran, A. Nurmi, R. Hodgson, Protein misfolding in neurodegenerative diseases: Implications and strategies. *Transl. Neurodegener.* **6**, 6 (2017).
2. C. J. Cortes, A. R. La Spada, TFEB dysregulation as a driver of autophagy dysfunction in neurodegenerative disease: Molecular mechanisms, cellular processes, and emerging therapeutic opportunities. *Neurobiol. Dis.* **122**, 83–93 (2019).
3. H. Martini-Stoica, A. L. Cole, D. B. Swartzlander, F. Chen, Y. W. Wan, L. Bajaj, D. A. Bader, V. M. Y. Lee, J. Q. Trojanowski, Z. Liu, M. Sardiello, H. Zheng, TFEB enhances astroglial uptake of extracellular tau species and reduces tau spreading. *J. Exp. Med.* **215**, 2355–2377 (2018).
4. Z. Gu, H. Cao, C. Zuo, Y. Huang, J. Miao, Y. Song, Y. Yang, L. Zhu, F. Wang, TFEB in Alzheimer's disease: From molecular mechanisms to therapeutic implications. *Neurobiol. Dis.* **173**, 105855 (2022).
5. X. He, Y. Xie, Q. Zheng, Z. Zhang, S. Ma, J. Li, M. Li, Q. Huang, TFE3-mediated autophagy is involved in dopaminergic neurodegeneration in Parkinson's disease. *Front. Cell Dev. Biol.* **9**, 761773 (2021).
6. M. P. Chan, A. A. Andea, P. W. Harms, A. B. Durham, R. M. Patel, M. Wang, P. Robichaud, G. J. Fisher, T. M. Johnson, D. R. Fullen, Genomic copy number analysis of a spectrum of blue nevi identifies recurrent aberrations of entire chromosomal arms in melanoma ex blue nevus. *Mod. Pathol.* **29**, 227–239 (2016).
7. J. Zou, B. C. Milon, M. M. Desouki, L. C. Costello, R. B. Franklin, hZIP1 zinc transporter down-regulation in prostate cancer involves the overexpression of ras responsive element binding protein-1 (RREB-1). *Prostate* **71**, 1518–1524 (2011).
8. M. Campa, M. Patel, P. Aubert, G. Hosler, D. Witheiler, Blue nevus–like metastasis of a cutaneous melanoma identified by fluorescence in situ hybridization. *Am. J. Dermatopathol.* **38**, 695–697 (2016).
9. G. Ferrara, A. C. De Vanna, Fluorescence in situ hybridization for melanoma diagnosis. *Am. J. Dermatopathol.* **38**, 253–269 (2016).
10. K. O'Hern, R. Barney, M. Chambers, C. Baker, M. Stevanovic, G. J. Tsongalis, E. Hughes, A. Sriharan, A novel method to assess copy number variation in melanoma: Droplet digital PCR for precise quantitation of the *RREB1* gene in FFPE melanocytic neoplasms, a proof-of-concept study. *J. Cutan. Pathol.* **50**, 169–177 (2022).
11. M. A. Pletneva, A. Andea, N. Palanisamy, B. L. Betz, S. Carskadon, M. Wang, R. M. Patel, D. R. Fullen, P. W. Harms, Clear cell melanoma: A cutaneous clear cell malignancy. *Arch. Pathol. Lab. Med.* **138**, 1328–1336 (2014).
12. J. Su, S. M. Morgani, C. J. David, Q. Wang, E. E. Er, Y.-H. Huang, H. Basnet, Y. Zou, W. Shu, R. K. Soni, R. C. Hendrickson, A.-K. Hadjantonakis, J. Massagué, TGF-β orchestrates fibrogenic and developmental EMTs via the RAS effector RREB1. *Nature* **577**, 566–571 (2020).
13. S. M. Morgani, J. Su, J. Nichols, J. Massagué, A.-K. Hadjantonakis, The transcription factor Rreb1 regulates epithelial architecture, invasiveness, and vasculogenesis in early mouse embryos. *eLife* **10**, e64811 (2021).
14. J. Oberdick, R. J. Smeyne, J. R. Mann, S. Zackson, J. I. Morgan, A promoter that drives transgene expression in cerebellar purkinje and retinal bipolar neurons. *Science* **248**, 223–226 (1990).
15. S. K. Ray, J. Nishitani, M. W. Petry, M. Y. Fessing, A. B. Leiter, Novel transcriptional potentiation of BETA2/NeuroD on the secretin gene promoter by the DNA-binding protein finb/RREB-1. *Mol. Cell. Biol.* **23**, 259–271 (2003).
16. E. Sanz, L. Yang, T. Su, D. R. Morris, G. S. McKnight, P. S. Amieux, Cell-type-specific isolation of ribosome-associated mRNA from complex tissues. *Proc. Natl. Acad. Sci. U.S.A.* **106**, 13939–13944 (2009).
17. Y. Liu, J. W. Lee, S. L. Ackerman, Mutations in the microtubule-associated protein 1A (Map1a) gene cause Purkinje cell degeneration. *J. Neurosci.* **35**, 4587–4598 (2015).
18. M. Ka, W.-Y. Kim, Microtubule-actin crosslinking factor 1 is required for dendritic arborization and axon outgrowth in the developing brain. *Mol. Neurobiol.* **53**, 6018–6032 (2016).
19. O. I. Kahn, P. Schätzle, D. van de Willige, R. P. Tas, F. W. Lindhout, S. Portegies, L. C. Kapitein, C. C. Hoogenraad, APC2 controls dendrite development by promoting microtubule dynamics. *Nat. Commun.* **9**, 2773 (2018).

20. D. Glick, S. Barth, K. F. Macleod, Autophagy: Cellular and molecular mechanisms. *J. Pathol.* **221**, 3–12 (2010).
21. C. Yang, X. Wang, Lysosome biogenesis: Regulation and functions. *J. Cell Biol.* **220**, e202102001 (2021).
22. T. Unno, M. Wakamori, M. Koike, Y. Uchiyama, K. Ishikawa, H. Kubota, T. Yoshida, H. Sasakawa, C. Peters, H. Mizusawa, K. Watase, Development of Purkinje cell degeneration in a knockin mouse model reveals lysosomal involvement in the pathogenesis of SCA6. *PNAS.* **109**, 17693–17698 (2012).
23. Y. R. Jo, H. R. Kim, S. Y. Jang, H. Go, M. Song, D. K. Park, Y. Oh, J. Jo, Y. K. Shin, S. J. Lee, S. Cheon, H. K. Lee, K. E. Lee, Y. H. Kim, H. T. Park, Potential neuron-autonomous Purkinje cell degeneration by 2′,3′-cyclic nucleotide 3′-phosphodiesterase promoter/ Cre-mediated autophagy impairments. *FASEB J.* **35**, e21225 (2021).
24. M. Koike, M. Shibata, T. Sunabori, J. Yamaguchi, K. Sakimura, M. Komatsu, K. Tanaka, Y. Uchiyama, Purkinje cells are more vulnerable to the specific depletion of cathepsin D than to that of Atg7. *Am. J. Pathol.* **187**, 1586–1600 (2017).
25. S. S. Mann, J. A. Hammarback, Molecular characterization of light chain 3. A microtubule binding subunit of MAP1A and MAP1B. *J. Biol. Chem.* **269**, 11492–11497 (1994).
26. J. Scheel, R. Matteoni, T. Ludwig, B. Hoflack, T. E. Kreis, Microtubule depolymerization inhibits transport of cathepsin D from the Golgi apparatus to lysosomes. *J. Cell Sci.* **96**, 711–720 (1990).
27. E. Fass, E. Shvets, I. Degani, K. Hirschberg, Z. Elazar, Microtubules support production of starvation-induced autophagosomes but not their targeting and fusion with lysosomes. *J. Biol. Chem.* **281**, 36303–36316 (2006).
28. A. Aplin, T. Jasionowski, D. L. Tuttle, S. E. Lenk, W. A. Dunn, Cytoskeletal elements are required for the formation and maturation of autophagic vacuoles. *J. Cell. Physiol.* **152**, 458–466 (1992).
29. R. Köchl, X. W. Hu, E. Y. W. Chan, S. A. Tooze, Microtubules facilitate autophagosome formation and fusion of autophagosomes with endosomes. *Traffic* **7**, 129–145 (2006).
30. J. L. Webb, B. Ravikumar, D. C. Rubinsztein, Microtubule disruption inhibits autophagosome-lysosome fusion: Implications for studying the roles of aggresomes in polyglutamine diseases. *Int. J. Biochem. Cell Biol.* **36**, 2541–2550 (2004).
31. M. Götz, W. B. Huttner, The cell biology of neurogenesis. *Nat. Rev. Mol. Cell Biol.* **6**, 777–788 (2005).
32. E. W. Dent, S. L. Gupton, F. B. Gertler, The growth cone cytoskeleton in axon outgrowth and guidance. *Cold Spring Harb. Perspect. Biol.* **3**, a001800 (2011).
33. C. Conde, A. Cáceres, Microtubule assembly, organization and dynamics in axons and dendrites. *Nat. Rev. Neurosci.* **10**, 319–332 (2009).
34. G. Kerjan, J. G. Gleeson, Genetic mechanisms underlying abnormal neuronal migration in classical lissencephaly. *Trends Genet.* **23**, 623–630 (2007).
35. M. A. Stouffer, J. A. Golden, F. Francis, Neuronal migration disorders: Focus on the cytoskeleton and epilepsy. *Neurobiol. Dis.* **92**, 18–45 (2016).
36. G. Szebenyi, F. Bollati, M. Bisbal, S. Sheridan, L. Faas, R. Wray, S. Haferkamp, S. Nguyen, A. Caceres, S. T. Brady, Activity-driven dendritic remodeling requires microtubule-associated protein 1A. *Curr. Biol.* **15**, 1820–1826 (2005).
37. P.-Y. Shih, S.-P. Lee, Y.-K. Chen, Y.-P. Hsueh, Cortactin binding protein 2 increases microtubule stability and regulates dendritic arborization. *J. Cell Sci.* **32**, 1043–1055 (2014).
38. K. K. Gupta, C. Li, A. Duan, E. O. Alberico, O. V. Kim, M. S. Alber, H. V. Goodson, Mechanism for the catastrophe-promoting activity of the microtubule destabilizer Op18/stathmin. *PNAS.* **110**, 20449–20454 (2013).
39. E. Charbaut, P. A. Curmi, S. Ozon, S. Lachkar, V. Redeker, A. Sobel, Stathmin family proteins display specific molecular and tubulin binding properties. *J. Biol. Chem.* **276**, 16146–16154 (2001).
40. N. Ohkawa, K. Fujitani, E. Tokunaga, S. Furuya, K. Inokuchi, The microtubule destabilizer stathmin mediates the development of dendritic arbors in neuronal cells. *J. Cell Sci.* **120**, 1447–1456 (2007).
41. F. E. Poulain, S. Chauvin, R. Wehrlé, M. Desclaux, J. Mallet, G. Vodjdani, I. Dusart, A. Sobel, SCLIP is crucial for the formation and development of the Purkinje cell dendritic arbor. *J. Neurosci.* **28**, 7387–7398 (2008).
42. W. Liedtke, E. E. Leman, R. E. W. Fyffe, C. S. Raine, U. K. Schubart, Stathmin-deficient mice develop an age-dependent axonopathy of the central and peripheral nervous systems. *Am. J. Pathol.* **160**, 469–480 (2002).
43. Z. Melamed, J. López-Erauskin, M. W. Baughn, O. Zhang, K. Drenner, Y. Sun, F. Freyermuth, M. A. McMahon, M. S. Beccari, J. W. Artates, T. Ohkubo, M. Rodriguez, N. Lin, D. Wu, C. F. Bennett, F. Rigo, S. Da Cruz, J. Ravits, C. Lagier-Tourenne, D. W. Cleveland, Premature polyadenylation-mediated loss of stathmin-2 is a hallmark of TDP-43-dependent neurodegeneration. *Nat. Neurosci.* **22**, 180–190 (2019).
44. J. D. Glass, Stathmin-2: Adding another piece to the puzzle of TDP-43 proteinopathies and neurodegeneration. *J. Clin. Investig.* **130**, 5677–5680 (2020).
45. O. A. Kent, M. Saha, E. Coyaud, H. E. Burston, N. Law, K. Dadson, S. Chen, E. M. Laurent, J. St-Germain, R. X. Sun, Y. Matsumoto, J. Cowen, A. Montgomery-Song, K. R. Brown, C. Ishak, J. La Rose, D. D. De Carvalho, H. H. He, B. Raught, F. Billia, P. Kannu, R. Rottapel, Haploinsufficiency of RREB1 causes a Noonan-like RASopathy via epigenetic reprogramming of RAS-MAPK pathway genes. *Nat. Commun.* **11**, 4673 (2020).
46. F. Lin, Z. V. Wang, J. A. Hill, Seeing is believing. *Autophagy* **10**, 691–693 (2014).
47. K. J. Livak, T. D. Schmittgen, Analysis of relative gene expression data using real-time quantitative PCR and the $2^{-\Delta\Delta CT}$ method. *Methods* **25**, 402–408 (2001).
48. L. Texari, N. J. Spann, T. D. Troutman, M. Sakai, J. S. Seidman, S. Heinz, An optimized protocol for rapid, sensitive and robust on-bead ChIP-seq from primary cells. *STAR Protoc.* **2**, 100358 (2021).
49. S. Chen, Y. Zhou, Y. Chen, J. Gu, fastp: An ultra-fast all-in-one FASTQ preprocessor. *Bioinformatics* **34**, i884–i890 (2018).
50. B. Langmead, S. L. Salzberg, Fast gapped-read alignment with Bowtie 2. *Nat. Methods* **9**, 357–359 (2012).
51. Y. Liao, G. K. Smyth, W. Shi, featureCounts: An efficient general purpose program for assigning sequence reads to genomic features. *Bioinformatics* **30**, 923–930 (2014).
52. M. I. Love, W. Huber, S. Anders, Moderated estimation of fold change and dispersion for RNA-seq data with DESeq2. *Genome Biol.* **15**, 550 (2014).
53. S. X. Ge, D. Jung, R. Yao, ShinyGO: A graphical gene-set enrichment tool for animals and plants. *Bioinformatics* **36**, 2628–2629 (2020).
54. A. Tarasov, A. J. Vilella, E. Cuppen, I. J. Nijman, P. Prins, Sambamba: Fast processing of NGS alignment formats. *Bioinformatics* **31**, 2032–2034 (2015).
55. Y. Zhang, T. Liu, C. A. Meyer, J. Eeckhoute, D. S. Johnson, B. E. Bernstein, C. Nusbaum, R. M. Myers, M. Brown, W. Li, X. S. Liu, Model-based analysis of ChIP-seq (MACS). *Genome Biol.* **9**, R137 (2008).
56. S. Heinz, C. Benner, N. Spann, E. Bertolino, Y. C. Lin, P. Laslo, J. X. Cheng, C. Murre, H. Singh, C. K. Glass, Simple combinations of lineage-determining transcription factors prime cis-regulatory elements required for macrophage and B cell identities. *Mol. Cell* **38**, 576–589 (2010).
57. X. Ran, P. Zhou, K. Zhang, Autophagy plays an important role in stemness mediation and the novel dual function of EIG121 in both autophagy and stemness regulation of endometrial carcinoma JEC cells. *Int. J. Oncol.* **51**, 644–656 (2017).
58. L. Deng, J. Feng, R. R. Broaddus, The novel estrogen-induced gene EIG121 regulates autophagy and promotes cell survival under stress. *Cell Death Dis.* **1**, e32–e32 (2010).
59. C. J. Hong, J. Yeon, B. K. Yeo, H. Woo, H. An, W. Heo, K. Kim, S. Yu, Fas-apoptotic inhibitory molecule 2 localizes to the lysosome and facilitates autophagosome-lysosome fusion through the LC3 interaction region motif–dependent interaction with LC3. *FASEB J.* **34**, 161–179 (2020).
60. X. Roa-Mansergas, R. Fadó, M. Atari, J. F. Mir, H. Muley, D. Serra, N. Casals, CPT1C promotes human mesenchymal stem cells survival under glucose deprivation through the modulation of autophagy. *Sci. Rep.* **8**, 6997 (2018).
61. I. S. Stein, A. Gottfried, J. Zimmermann, G. Fischer von Mollard, TVP23interacts genetically with the yeast SNAREVTI1and functions in retrograde transport from the early endosome to the late Golgi. *Biochem. J.* **419**, 229–236 (2009).
62. W. Wang, Z. Xia, J.-C. Farré, S. Subramani, TRIM37 deficiency induces autophagy through deregulating the MTORC1-TFEB axis. *Autophagy* **14**, 1574–1585 (2018).
63. Y. Wen, R. G. Zhai, M. D. Kim, The role of autophagy in Nmnat-mediated protection against hypoxia-induced dendrite degeneration. *Mol. Cell. Neurosci.* **52**, 140–151 (2013).
64. A. K. H. Stavoe, S. E. Hill, D. H. Hall, D. A. Colón-Ramos, KIF1A/UNC-104 transports ATG-9 to regulate neurodevelopment and autophagy at synapses. *Dev. Cell* **38**, 171–185 (2016).
65. D. W. Lawrence, P. A. Willard, A. M. Cochran, E. C. Matchett, J. Kornbluth, Natural killer lytic-associated molecule (NKLAM): An E3 ubiquitin ligase with an integral role in innate immunity. *Front. Physiol.* **11**, 573372 (2020).
66. F. Nieto-Jacobo, D. Pasch, C. W. Basse, The mitochondrial Dnm1-Like fission component is required for lga2 -induced mitophagy but dispensable for starvation-induced mitophagy in ustilago maydis. *Eukaryot. Cell* **11**, 1154–1166 (2012).
67. M. Chen, Z. Chen, Y. Wang, Z. Tan, C. Zhu, Y. Li, Z. Han, L. Chen, R. Gao, L. Liu, Q. Chen, Mitophagy receptor FUNDC1 regulates mitochondrial dynamics and mitophagy. *Autophagy* **12**, 689–702 (2016).
68. C.-M. Xie, Y. Sun, The MTORC1-mediated autophagy is regulated by the FBXW7-SHOC2-RPTOR axis. *Autophagy* **15**, 1470–1472 (2019).
69. Y. Sun, W. Nie, B. Qiu, Q. Yang, H. Zhao, FBXW7 affects autophagy through MCL1 in oral squamous cell carcinoma. *Oral Dis.* **29**, 3259–3267 (2022).
70. L. Popineau, L. Morzyglod, N. Carré, M. Caüzac, P. Bossard, C. Prip-Buus, V. Lenoir, B. Ragazzon, V. Fauveau, L. Robert, S. Guilmeau, C. Postic, M. Komatsu, F. Canonne-Hergaux, H. Guillou, A.-F. Burnol, Novel Grb14-mediated cross talk between insulin and p62/Nrf2 pathways regulates liver lipogenesis and selective insulin resistance. *Mol. Cell. Biol.* **36**, 2168–2181 (2016).
71. E. Zavodszky, M. N. J. Seaman, K. Moreau, M. Jimenez-Sanchez, S. Y. Breusegem, M. E. Harbour, D. C. Rubinsztein, Mutation in VPS35 associated with Parkinson's disease impairs WASH complex association and inhibits autophagy. *Nat. Commun.* **5**, 3828 (2014).
72. K. Kato, Y. Oka, H. Muramatsu, F. F. Vasilev, T. Otomo, H. Oishi, Y. Kawano, H. Kidokoro, Y. Nakazawa, T. Ogi, Y. Takahashi, S. Saitoh, Biallelic VPS35L pathogenic variants cause 3C/

Ritscher-Schinzel-like syndrome through dysfunction of retriever complex. *J. Med. Genet.* **57**, 245–253 (2020).
73. K. Jiang, M. Liu, G. Lin, B. Mao, W. Cheng, H. Liu, J. Gal, H. Zhu, Z. Yuan, W. Deng, Q. Liu, P. Gong, X. Bi, S. Meng, Tumor suppressor Spred2 interaction with LC3 promotes autophagosome maturation and induces autophagy-dependent cell death. *Oncotarget* **7**, 25652–25667 (2016).
74. M. Ullrich, B. Aßmus, A. M. Augustin, H. Häbich, M. Abeßer, J. Martin Machado, F. Werner, R. Erkens, A.-P. Arias-Loza, S. Umbenhauer, H. Wagner, P. M. Benz, A. Unger, W. A. Linke, S. Frantz, H. A. Baba, M. Kuhn, K. Schuh, SPRED2 deficiency elicits cardiac arrhythmias and premature death via impaired autophagy. *J. Mol. Cell. Cardiol.* **129**, 13–26 (2019).
75. C. Xie, S. Liu, B. Wu, Y. Zhao, B. Chen, J. Guo, S. Qiu, Y. Cao, miR-19 promotes cell proliferation, invasion, migration, and emt by inhibiting SPRED2-mediated autophagy in osteosarcoma cells. *Cell Transplant.* **29**, 963689720962460 (2020).
76. X. Liang, C. Wang, Y. Sun, W. Song, J. Lin, J. Li, X. Guan, p62/mTOR/LXRα pathway inhibits cholesterol efflux mediated by ABCA1 and ABCG1 during autophagy blockage. *Biochem. Biophys. Res. Commun.* **514**, 1093–1100 (2019).
77. S. L. Prosser, J. Tkach, L. Gheiratmand, J. Kim, B. Raught, C. G. Morrison, L. Pelletier, Aggresome assembly at the centrosome is driven by CP110–CEP97–CEP290 and centriolar satellites. *Nat. Cell Biol.* **24**, 483–496 (2022).
78. S. Imai, N. Okayama, M. Shimizu, M. Itoh, Increased intracellular calcium activates serum and glucocorticoid-inducible kinase 1 (SGK1) through a calmodulin-calcium calmodulin dependent kinase kinase pathway in Chinese hamster ovary cells. *Life Sci.* **72**, 2199–2209 (2003).
79. O. Borst, E.-M. Schmidt, P. Münzer, T. Schönberger, S. T. Towhid, M. Elvers, C. Leibrock, E. Schmid, A. Eylenstein, D. Kuhl, A. E. May, M. Gawaz, F. Lang, The serum- and glucocorticoid-inducible kinase 1 (SGK1) influences platelet calcium signaling and function by regulation of Orai1 expression in megakaryocytes. *Blood* **119**, 251–261 (2012).
80. T. R. H. Jouen-Tachoire, S. J. Tucker, P. Tammaro, Ion channels as convergence points in the pathology of pulmonary arterial hypertension. *Biochem. Soc. Trans.* **49**, 1855–1865 (2021).
81. H. V. McCue, R. D. Burgoyne, L. P. Haynes, Membrane targeting of the EF-hand containing calcium-sensing proteins CaBP7 and CaBP8. *Biochem. Biophys. Res. Commun.* **380**, 825–831 (2009).
82. F. Haeseleer, I. Sokal, C. L. M. J. Verlinde, H. Erdjument-Bromage, P. Tempst, A. N. Pronin, J. L. Benovic, R. N. Fariss, K. Palczewski, Five members of a novel Ca2+−binding protein (CABP) subfamily with similarity to calmodulin. *J. Biol. Chem.* **275**, 1247–1260 (2000).
83. P. H. Cheung, W. Luo, Y. Qiu, X. Zhang, K. Earley, P. Millirons, S. H. Lin, Structure and function of C-CAM1. The first immunoglobulin domain is required for intercellular adhesion. *J. Biol. Chem.* **268**, 24303–24310 (1993).
84. W. Luo, C. G. Wood, K. Earley, M.-C. Hung, S.-H. Lin, Suppression of tumorigenicity of breast cancer cells by an epithelial cell adhesion molecule (C-CAM1): The adhesion and growth suppression are mediated by different domains. *Oncogene* **14**, 1697–1704 (1997).
85. M. Terashima, M. Kobayashi, M. Motomiya, N. Inoue, T. Yoshida, H. Okano, N. Iwasaki, A. Minami, I. Matsuoka, Analysis of the expression and function of BRINP family genes during neuronal differentiation in mouse embryonic stem cell-derived neural stem cells. *J. Neurosci. Res.* **88**, 1387–1393 (2010).
86. S. R. Berkowicz, T. J. Featherby, Z. Qu, A. Giousoh, N. A. Borg, J. I. Heng, J. C. Whisstock, P. I. Bird, Brinp1$^{-/-}$ mice exhibit autism-like behaviour, altered memory, hyperactivity and increased parvalbumin-positive cortical interneuron density. *Mol. Autism.* **7**, 22 (2016).
87. M. Hermann, R. Reumann, K. Schostak, D. Kement, M. Gelderblom, C. Bernreuther, R. Frischknecht, A. Schipanski, S. Marik, S. Krasemann, D. Sepulveda-Falla, M. Schweizer, T. Magnus, M. Glatzel, G. Galliciotti, Deficits in developmental neurogenesis and dendritic spine maturation in mice lacking the serine protease inhibitor neuroserpin. *Mol. Cell. Neurosci.* **102**, 103420 (2020).
88. R. Reumann, R. Vierk, L. Zhou, F. Gries, V. Kraus, J. Mienert, E. Romswinkel, F. Morellini, I. Ferrer, C. Nicolini, M. Fahnestock, G. Rune, M. Glatzel, G. Galliciotti, The serine protease inhibitor neuroserpin is required for normal synaptic plasticity and regulates learning and social behavior. *Learn. Mem.* **24**, 650–659 (2017).
89. J. Kashef, T. Diana, M. Oelgeschläger, I. Nazarenko, Expression of the tetraspanin family members Tspan3, Tspan4, Tspan5 and Tspan7 during *Xenopus laevis* embryonic development. *Gene Expr. Patterns* **13**, 1–11 (2013).
90. J. Xiao, R. Dai, L. Negyessy, C. Bergson, Calcyon, a novel partner of clathrin light chain, stimulates clathrin-mediated endocytosis. *J. Biol. Chem.* **281**, 15182–15193 (2006).
91. M. Kruusmägi, S. Zelenin, H. Brismar, L. Scott, Intracellular dynamics of calcyon, a neuron-specific vesicular protein. *Neuroreport* **18**, 1547–1551 (2007).
92. S.-E. Lee, S. Jeong, U. Lee, S. Chang, SGIP1α functions as a selective endocytic adaptor for the internalization of synaptotagmin 1 at synapses. *Mol. Brain* **12**, 41 (2019).
93. A. Uezu, A. Horiuchi, K. Kanda, N. Kikuchi, K. Umeda, K. Tsujita, S. Suetsugu, N. Araki, H. Yamamoto, T. Takenawa, H. Nakanishi, SGIP1α is an endocytic protein that directly interacts with phospholipids and Eps15. *J. Biol. Chem.* **282**, 26481–26489 (2007).
94. B. Ritter, S. Murphy, H. Dokainish, M. Girard, M. V. Gudheti, G. Kozlov, M. Halin, J. Philie, E. M. Jorgensen, K. Gehring, P. S. McPherson, NECAP 1 regulates AP-2 interactions to control vesicle size, number, and cargo during clathrin-mediated endocytosis. *PLoS Biol.* **11**, e1001670 (2013).
95. K. Goto, H. Kondo, Diacylglycerol kinase in the central nervous system—Molecular heterogeneity and gene expression. *Chem. Phys. Lipids* **98**, 109–117 (1999).
96. L. Wang, L. Zhu, C. C. Wang, The endoplasmic reticulum sulfhydryl oxidase Ero1β drives efficient oxidative protein folding with loose regulation. *Biochem. J.* **434**, 113–121 (2011).
97. M. Pagani, M. Fabbri, C. Benedetti, A. Fassio, S. Pilati, N. J. Bulleid, A. Cabibbo, R. Sitia, Endoplasmic reticulum oxidoreductin 1-Lβ (ERO1-Lβ), a human gene induced in the course of the unfolded protein response. *J. Biol. Chem.* **275**, 23685–23692 (2000).
98. M. W. Breuss, A. Nguyen, Q. Song, T. Nguyen, V. Stanley, K. N. James, D. Musaev, G. Chai, S. A. Wirth, P. Anzenberg, R. D. George, A. Johansen, S. Ali, M. Zia-ur-Rehman, T. Sultan, M. S. Zaki, J. G. Gleeson, Mutations in LNPK, encoding the endoplasmic reticulum junction stabilizer lunapark, cause a recessive neurodevelopmental syndrome. *Am. J. Hum. Genet.* **103**, 296–304 (2018).
99. A. Tsuchida, M. Ogiso, Y. Nakamura, M. Kiso, K. Furukawa, K. Furukawa, Molecular cloning and expression of human ST6GalNAc III: Restricted tissue distribution and substrate specificity. *J. Biochem.* **138**, 237–243 (2005).
100. Z. Chen, Z. G. Gulzar, C. A. St. Hill, B. Walcheck, J. D. Brooks, Increased expression of GCNT1 is associated with altered O-glycosylation of PSA, PAP, and MUC1 in human prostate cancers. *Prostate* **74**, 1059–1067 (2014).
101. E. Perkey, D. Maurice De Sousa, L. Carrington, J. Chung, A. Dils, D. Granadier, U. Koch, F. Radtke, B. Ludewig, B. R. Blazar, C. W. Siebel, T. V. Brennan, J. Nolz, N. Labrecque, I. Maillard, GCNT1-mediated O-glycosylation of the sialomucin CD43 is a sensitive indicator of notch signaling in activated T cells. *J. Immunol.* **204**, 1674–1688 (2020).
102. X. Li, J. Wang, W. Li, Y. Xu, D. Shao, Y. Xie, W. Xie, T. Kubota, H. Narimatsu, Y. Zhang, Characterization of ppGalNAc-T18, a member of the vertebrate-specific Y subfamily of UDP-N-acetyl-α-D-galactosamine:polypeptide N-acetylgalactosaminyltransferases. *Glycobiology* **22**, 602–615 (2012).
103. N. Naslavsky, J. McKenzie, N. Altan-Bonnet, D. Sheff, S. Caplan, EHD3 regulates early-endosome-to-Golgi transport and preserves Golgi morphology. *J. Cell Sci.* **122**, 389–400 (2009).
104. K. Bahl, S. Xie, G. Spagnol, P. Sorgen, N. Naslavsky, S. Caplan, EHD3 protein is required for tubular recycling endosome stabilization, and an asparagine-glutamic acid residue pair within its Eps15 homology (EH) domain dictates its selective binding to NPF peptides. *J. Biol. Chem.* **291**, 13465–13478 (2016).
105. T. K. Bjarnadóttir, R. Fredriksson, H. B. Schiöth, The gene repertoire and the common evolutionary history of glutamate, pheromone (V2R), taste(1) and other related G protein-coupled receptors. *Gene* **362**, 70–84 (2005).
106. J. Sheng, J. Xu, K. Geng, D. Liu, Sema6D regulates zebrafish vascular patterning and motor neuronal axon growth in spinal cord. *Front Mol Neurosci.* **15**, 854556 (2022).
107. A. Garbouchian, A. C. Montgomery, S. P. Gilbert, M. Bentley, KAP is the neuronal organelle adaptor for kinesin-2 KIF3AB and KIF3AC. *Mol. Biol. Cell* **33**, ar133 (2022).
108. H. Yamazaki, T. Nakata, Y. Okada, N. Hirokawa, Cloning and characterization of KAP3: A novel kinesin superfamily-associated protein of KIF3A/3B (microtubule/axonal transport/motor protein). *Cell Biol.* **93**, 8443–8448 (1996).
109. M. A. Felmlee, R. S. Jones, V. Rodriguez-Cruz, K. E. Follman, M. E. Morris, Monocarboxylate transporters (SLC16): Function, regulation, and role in health and disease. *Pharmacol. Rev.* **72**, 466–485 (2020).
110. S. Poliak, L. Gollan, R. Martinez, A. Custer, S. Einheber, J. L. Salzer, J. S. Trimmer, P. Shrager, E. Peles, Caspr2, a new member of the neurexin superfamily, is localized at the juxtaparanodes of myelinated axons and associates with k channels. *Neuron* **24**, 1037–1047 (1999).
111. F. St George-Hyslop, M. Haneklaus, T. Kivisild, F. J. Livesey, Loss of CNTNAP2 alters human cortical excitatory neuron differentiation and neural network development. *Biol. Psychiatry* **94**, 780–791 (2023).
112. M. Poot, Connecting the CNTNAP2 networks with neurodevelopmental disorders. *Mol Syndromol.* **6**, 7–22 (2015).
113. M. Gaitanou, K. Segklia, R. Matsas, Cend1, a story with many tales: From regulation of cell cycle progression/exit of neural stem cells to brain structure and function. *Stem Cells Int.* **2019**, **2019**, 1–16
114. A. I. R. Spanjer, H. A. Baarsma, L. M. Oostenbrink, S. R. Jansen, C. C. Kuipers, M. Lindner, D. S. Postma, H. Meurs, I. H. Heijink, R. Gosens, M. Königshoff, A. M. Fachkliniken, TGF-b-induced profibrotic signaling is regulated in part by the WNT receptor Frizzled-8. *J (Basel).* **30**, 1823–1835 (2016).
115. K. Itoh, J. Jacob, S. Y. Sokol, A role for xenopus Frizzled 8 in dorsal development. *Mech. Dev.* **74**, 145–157 (1998).
116. M. A. Deardorff, C. Tan, L. J. Conrad, P. S. Klein, Frizzled-8 is expressed in the Spemann organizer and plays a role in early morphogenesis. *Development* **125**, 2687–2700 (1998).
117. D. J. Gillooly, A. Simonsen, H. Stenmark, Cellular functions of phosphatidylinositol 3-phosphate and FYVE domain proteins. *Biochem. J.* **355**, 249–258 (2001).
118. N. J. Blunsom, S. Cockcroft, CDP-diacylglycerol synthases (CDS): Gateway to phosphatidylinositol and cardiolipin synthesis. *Front. Cell Dev. Biol.* **8**, 63 (2020).

119. P.-L. Hsu, J. Jou, S.-J. Tsai, TYRO3: A potential therapeutic target in cancer. *Exp. Biol. Med.* **244**, 83–99 (2019).
120. S. Chauvin, A. Sobel, Neuronal stathmins: A family of phosphoproteins cooperating for neuronal development, plasticity and regeneration. *Prog. Neurobiol.* **126**, 1–18 (2015).
121. V. C. Foletta, N. Moussi, P. D. Sarmiere, J. R. Bamburg, O. Bernard, LIM kinase 1, a key regulator of actin dynamics, is widely expressed in embryonic and adult tissues. *Exp. Cell Res.* **294**, 392–405 (2004).
122. D. C. Muñoz-Lasso, C. Romá-Mateo, F. V. Pallardó, P. Gonzalez-Cabo, Much more than a Scaffold: Cytoskeletal proteins in neurological disorders. *Cell* **9**, 358 (2020).
123. C. Y. Lin, H. L. Lai, H. M. Chen, J. J. Siew, C. Te Hsiao, H. C. Chang, K. S. Liao, S. C. Tsai, C. Y. Wu, K. Kitajima, C. Sato, K. H. Khoo, Y. Chern, Functional roles of ST8SIA3-mediated sialylation of striatal dopamine D2 and adenosine A2A receptors. *Transl. Psychiatry* **2019**. **9**, 1–13 (**2019**).
124. R.-B. Huang, D. Cheng, S.-M. Liao, B. Lu, Q.-Y. Wang, N.-Z. Xie, F. A. Troy II, G.-P. Zhou, The intrinsic relationship between structure and function of the sialyltransferase ST8Sia family members. *Curr. Top. Med. Chem.* **17**, 2359–2369 (2017).
125. J. Liu, X. Zheng, X. Pang, L. Li, J. Wang, C. Yang, G. Du, Ganglioside GD3 synthase (GD3S), a novel cancer drug target. *Acta Pharm. Sin.* **8**, 713–720 (2018).
126. E. R. Sturgill, K. Aoki, P. Hh Lopez, D. Colacurcio, K. Vajn, I. Lorenzini, S. Majić, W. H. Yang, M. Heffer, M. Tiemeyer, J. D. Marth, R. L. Schnaar, Biosynthesis of the major brain gangliosides GD1a and GT1b. *Glycobiology* **22**, 1289–1301 (2012).
127. S. W. Yoo, M. G. Motari, K. Susuki, J. Prendergast, A. Mountney, A. Hurtado, R. L. Schnaar, Sialylation regulates brain structure and function. *FASEB J.* **29**, 3040–3053 (2015).
128. N. W. Shworak, J. Liu, L. M. Petros, L. Zhang, M. Kobayashi, N. G. Copeland, N. A. Jenkins, R. D. Rosenberg, Multiple isoforms of heparan sulfate D-glucosaminyl 3-O-sulfotransferase: Isolation, characterization, and expression of human cdnas and identification of distinct genomic loci. *J. Biol. Chem.* **274**, 5170–5184 (1999).
129. H. Ota, S. Nishihara, Regulation of 3-O-sulfation of heparan sulfate during transition from the naïve to the primed state in mouse embryonic stem cells. *Methods Mol. Biol.* **2303**, 443–452 (2022).
130. N. Kaempf, G. Kochlamazashvili, D. Puchkov, T. Maritzen, S. M. Bajjalieh, N. L. Kononenko, V. Haucke, Overlapping functions of stonin 2 and SV2 in sorting of the calcium sensor synaptotagmin 1 to synaptic vesicles. *Proc. Natl. Acad. Sci. U.S.A.* **112**, 7297–7302 (2015).
131. A. Nowack, J. Yao, K. L. Custer, S. M. Bajjalieh, SV2 regulates neurotransmitter release via multiple mechanisms. *Am. J. Physiol. Cell Physiol.* **299**, 960–967 (2010).
132. V. Rossi, R. Picco, M. Vacca, M. D'Esposito, M. D'Urso, T. Galli, F. Filippini, VAMP subfamilies identified by specific R-SNARE motifs. *Biol. Cell* **96**, 251–256 (2004).
133. Y. Wu, H. Matsui, K. Tomizawa, Amphiphysin I and regulation of synaptic vesicle endocytosis. *Acta Med. Okayama* **63**, 305–323 (2009).
134. C. David, P. S. McPherson, O. Mundigl, P. De Camilli, A role of amphiphysin in synaptic vesicle endocytosis suggested by its binding to dynamin in nerve terminals. *PNAS.* **93**, 331–335 (1996).
135. F. Antonucci, I. Corradini, G. Fossati, R. Tomasoni, E. Menna, M. Matteoli, SNAP-25, a known presynaptic protein with emerging postsynaptic functions. *Front Synaptic Neurosci.* **8**, 187218 (2016).
136. S. Sugita, R. Janz, T. C. Südhof, Synaptogyrins regulate Ca2+–dependent exocytosis in PC12 Cells. *J. Biol. Chem.* **274**, 18893–18901 (1999).
137. R. Mohrmann, H. de Wit, E. Connell, P. S. Pinheiro, C. Leese, D. Bruns, B. Davletov, M. Verhage, J. B. Sørensen, Synaptotagmin interaction with SNAP-25 governs vesicle docking, priming, and fusion triggering. *J. Neurosci.* **33**, 14417–14430 (2013).
138. R. G. Whittaker, D. N. Herrmann, B. Bansagi, B. A. S. Hasan, R. M. Lofra, E. L. Logigian, J. E. Sowden, J. L. Almodovar, J. T. Littleton, S. Zuchner, R. Horvath, H. Lochmüller, Electrophysiologic features of SYT2 mutations causing a treatable neuromuscular syndrome. *Neurology* **85**, 1964–1971 (2015).



**Acknowledgments:** We thank S. Adamson and C. Guzman for assistance with sequencing data; G. Reeves and N. Pongsamart for technical assistance; T. Kodama at the Du Lac laboratory for sharing the PCP2 promoter construct; J. Zhao and the transgenic mouse core at UCSD for pronuclear injection and transgenic mouse production; the UCSD School of Medicine Microscopy Core; the UCSD Nikon Imaging Center for use of equipment; and the UC San Diego IGM Genomics Center for use of an Illumina NovaSeq 6000. S.L.A. is an investigator of the Howard Hughes Medical Institute. **Funding:** This work was supported by National Institutes of Health grant R24 0D021325 (B.S.H.), National Institutes of Health grant T32GM008666 (E.N.G.), National Institutes of Health grant F31NS117131 (E.N.G.), Howard Hughes Medical Institute (S.L.A.), NINDS grant P30NS047101 (UCSD School of Medicine Microscopy Core), NIH SIG grant S10 OD026929 (UCSD IGM Genomics Center), and National Institutes of Health grant R01GM129523 (S.H.). **Author contributions:** Mutant identification: B.S.H. Conceptualization: S.L.A. Mouse colony maintenance: E.N.G., T.J., and B.S.H. Experimental design: E.N.G. and S.L.A. Provided ChIP-seq protocol: S.H. Performed experiments: E.N.G., T.J., and S.-E.S. Data interpretation: E.N.G. and S.L.A. Writing—original draft: E.N.G. and S.L.A. Writing—review and editing: E.N.G. and S.L.A. Research supervision: S.L.A. **Competing interests:** The authors declare that they have no competing interests. **Data and materials availability:** The datasets generated during this study have been deposited at the GEO database with the ID GSE223881. All data needed to evaluate the conclusions in the paper are present in the paper and/or the Supplementary Materials.

Submitted 2 March 2023
Accepted 12 December 2023
Published 10 January 2024
10.1126/sciadv.adh3929
