# Supplementary material for: RREB1 regulates neuronal proteostasis and the microtubule network
Source: Sci Adv. 2024 Jan 10;10(2):eadh3929. doi: 10.1126/sciadv.adh3929 (PMC10780896; doi:10.1126/sciadv.adh3929)
Supplement: Supplementary file 2 — Figs. S1 and S2 Legend for data S1 Legends for movies S1 and S2 References [file sciadv.adh3929_sm.pdf]

Science Advances
AAAS

Supplementary Materials for

RREB1 regulates neuronal proteostasis and the microtubule network

Emily N. Griffin *et al.*

Corresponding author: Susan L. Ackerman, sackerman@ucsd.edu

Sci. Adv. **10**, eadh3929 (2024)
DOI: 10.1126/sciadv.adh3929

The PDF file includes:

Figs. S1 and S2
Legend for data S1
Legends for movies S1 and S2
References

Other Supplementary Material for this manuscript includes the following:

Data S1
Movies S1 and S2

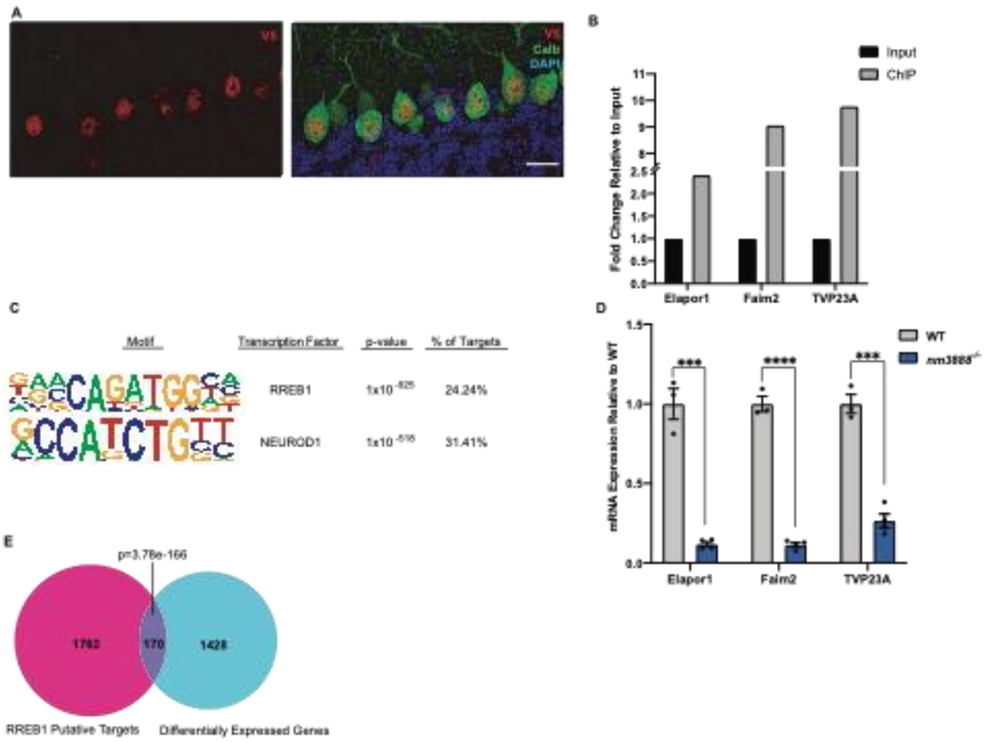

Fig. S1. The RREB1 V7 transgene localizes to Purkinje cell nuclei and binds target genes. (A) Immunofluorescence with antibodies for V5 and calbindin D-28 (Calb) in the cerebellum of a 6-week-old *Tg(PCP2-Rreb1/3xV5)20/J* mouse. The section was counterstained with DAPI. Scale bar is 25µm. (**B**) *De novo* motif enrichment for RREB1 in ChIP peaks relative to input peaks, and known motif enrichment for NEUROD1, a known binding partner of RREB1. (**C**) Venn diagram of the overlap between putative RREB1 Purkinje cell targets identified by ChIP-seq, and genes differentially expressed in *nm3888*$^{-/-}$ Purkinje cells relative to wild-type, identified by RNA-seq. A hypergeometric test was performed for the overlap and the P-value is indicated on the diagram. (**D**) ChIP-qPCR performed on pooled samples for three RREB1 targets identified in ChIP-seq experiment. (**E**) RT-qPCR performed on wild-type and *nm3888*$^{-/-}$ Purkinje cell transcripts for three significantly differentially expressed genes identified in RNA-seq experiment.

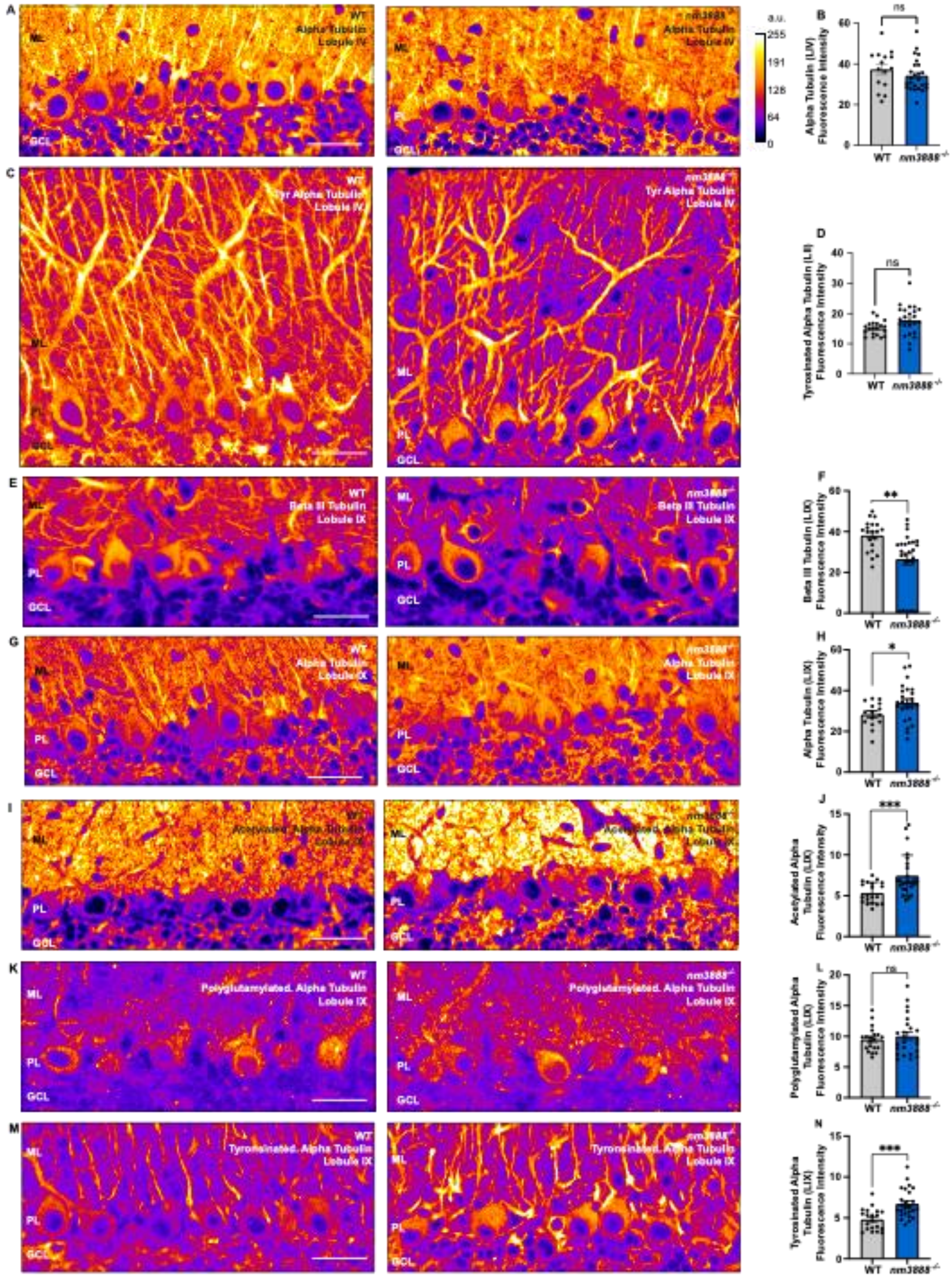

Fig. S2. Tubulin and associated posttranslational modifications vary in the rostral and caudal region of *nm3888*$^{-/-}$ Purkinje cells, relative to wild-type. (**A**) Immunofluorescence with antibodies to alpha tubulin in lobule IV and (**C**) Immunofluorescence with antibodies to tyrosinated alpha tubulin in lobule II. (**E**, **G**, **I**, **K**, **M**) Immunofluorescence lobule IX with antibodies to alpha tubulin (**E**), beta III tubulin (**G**), acetylated alpha tubulin (**I**), polyglutamylated alpha tubulin (**K**) and tyrosinated alpha tubulin (**M**). (**B**, **D**, **F**, **H**, **J**, **L**, **N**) Average cell soma fluorescence intensity with SEM represented by error bars. We performed multiple t-tests, and Bonferroni adjusted p-values are indicated (*) P≤ 0.05, (**) P≤0.01, (***) P≤0.001, (ns) non-significant. (**A**, **C**, **E**, **G**, **I**, **K**, **M**) Images are

pseudocolored based on intensity, an 8-bit LUT scale ranging from 0 to 255 was used. Scale bars are 25µm. (**A**-**N**) We used three-week-old mice for images and quantification.

Data S1. Results from analyses of RNA and ChIP seq experiments. (external file)

(**RNA_seq_DE**) Contains statistics from DESeq2 analysis of wild-type and *nm3888*$^{-/-}$ Purkinje cell RNA-seq data as well as raw read counts for each sample analyzed. (**GO_Mol_Path_Upreg**) Contains results from ShinyGO molecular pathway analysis run on all significantly upregulated genes (P-adj≤0.05). (**GO_Mol_Path_Downreg**) Contains results from ShinyGO molecular pathway analysis run on all significantly downregulated genes (P-adj≤0.05). (**ChIPseq_RNAseq_Overlap**) Contains genes that both contained RREB1 peaks defined in our ChIP-seq analysis and were significantly differentially expressed in our RNA-seq analysis of wild-type and *nm3888*$^{-/-}$ Purkinje cells (P-adj≤0.05). Each of these gene contains corresponding information from DESeq2 analysis. (**ChIP_seq**) Contains results from HOMER-annotated peaks called with MACS3. (**ChIP_Cell_Comp**) Contains results from ShinyGO cellular component enrichment analysis for all genes containing RREB1 peaks as defined by our ChIP-seq analysis. (**Endo_Targets**) Contains ChIP-seq-defined RREB1 targets belonging to the endomembrane system that are significantly differentially expressed between wild-type and *nm3888*$^{-/-}$ Purkinje cells (P-adj≤0.05). We manually grouped targets into one of 24 functional categories based on known molecular functions.

Supplement Video 1. Ataxia in 4-week-old *nm3888*$^{-/-}$ mice. (external file)

Two 4-week-old mice are shown from a top view. In the first frame, the wild-type mouse is at the top of the screen and the *nm3888*$^{-/-}$ mouse is beneath it.

Supplement Video 2. Ataxia in 11-month-old *nm3888*$^{-/-}$ mice. (external file)

Two 11-month-old mice are shown from a top view. In the first frame, the wild-type mouse is in the upper left corner of the screen and the *nm3888*$^{-/-}$ mouse is in the bottom center of the screen.

REFERENCES AND NOTES

1. P. Sweeney, H. Park, M. Baumann, J. Dunlop, J. Frydman, R. Kopito, A. McCampbell, G. Leblanc, A. Venkateswaran, A. Nurmi, R. Hodgson, Protein misfolding in neurodegenerative diseases: Implications and strategies. *Transl. Neurodegener.* **6**, 6 (2017).

2. C. J. Cortes, A. R. La Spada, TFEB dysregulation as a driver of autophagy dysfunction in neurodegenerative disease: Molecular mechanisms, cellular processes, and emerging therapeutic opportunities. *Neurobiol. Dis.* **122** 83–93 (2019).

3. H. Martini-Stoica, A. L. Cole, D. B. Swartzlander, F. Chen, Y. W. Wan, L. Bajaj, D. A. Bader, V. M. Y. Lee, J. Q. Trojanowski, Z. Liu, M. Sardiello, H. Zheng, TFEB enhances astroglial uptake of extracellular tau species and reduces tau spreading. *J. Exp. Med.* **215**, 2355–2377 (2018).

4. Z. Gu, H. Cao, C. Zuo, Y. Huang, J. Miao, Y. Song, Y. Yang, L. Zhu, F. Wang, TFEB in Alzheimer's disease: From molecular mechanisms to therapeutic implications. *Neurobiol. Dis.* **173**, 105855 (2022).

5. X. He, Y. Xie, Q. Zheng, Z. Zhang, S. Ma, J. Li, M. Li, Q. Huang, TFE3-mediated autophagy is involved in dopaminergic neurodegeneration in Parkinson's disease. *Front. Cell Dev. Biol.* **9**, 761773 (2021).

6. M. P. Chan, A. A. Andea, P. W. Harms, A. B. Durham, R. M. Patel, M. Wang, P. Robichaud, G. J. Fisher, T. M. Johnson, D. R. Fullen, Genomic copy number analysis of a spectrum of blue nevi identifies recurrent aberrations of entire chromosomal arms in melanoma ex blue nevus. *Mod. Pathol.* **29**, 227–239 (2016).

7. J. Zou, B. C. Milon, M. M. Desouki, L. C. Costello, R. B. Franklin, hZIP1 zinc transporter down-regulation in prostate cancer involves the overexpression of ras responsive element binding protein-1 (RREB-1). *Prostate* **71**, 1518–1524 (2011).

8. M. Campa, M. Patel, P. Aubert, G. Hosler, D. Witheiler, Blue nevus–like metastasis of a cutaneous melanoma identified by fluorescence in situ hybridization. *Am. J. Dermatopathol.* **38**, 695–697 (2016).

9. G. Ferrara, A. C. De Vanna, Fluorescence in situ hybridization for melanoma diagnosis. *Am. J. Dermatopathol.* **38**, 253–269 (2016).

10. K. O'Hern, R. Barney, M. Chambers, C. Baker, M. Stevanovic, G. J. Tsongalis, E. Hughes, A. Sriharan, A novel method to assess copy number variation in melanoma: Droplet digital PCR for precise quantitation of the *RREB1* gene in FFPE melanocytic neoplasms, a proof-of-concept study. *J. Cutan. Pathol.* **50**, 169–177 (2022).

11. M. A. Pletneva, A. Andea, N. Palanisamy, B. L. Betz, S. Carskadon, M. Wang, R. M. Patel, D. R. Fullen, P. W. Harms, Clear cell melanoma: A cutaneous clear cell malignancy. *Arch. Pathol. Lab. Med.* **138**, 1328–1336 (2014).

12. J. Su, S. M. Morgani, C. J. David, Q. Wang, E. E. Er, Y.-H. Huang, H. Basnet, Y. Zou, W. Shu, R. K. Soni, R. C. Hendrickson, A.-K. Hadjantonakis, J. Massagué, TGF-β orchestrates fibrogenic and developmental EMTs via the RAS effector RREB1. *Nature* **577**, 566–571 (2020).

13. S. M. Morgani, J. Su, J. Nichols, J. Massagué, A.-K. Hadjantonakis, The transcription factor Rreb1 regulates epithelial architecture, invasiveness, and vasculogenesis in early mouse embryos. *eLife* **10,** e64811 (2021).

14. J. Oberdick, R. J. Smeyne, J. R. Mann, S. Zackson, J. I. Morgan, A promoter that drives transgene expression in cerebellar purkinje and retinal bipolar neurons. *Science* **248**, 223–226 (1990).

15. S. K. Ray, J. Nishitani, M. W. Petry, M. Y. Fessing, A. B. Leiter, Novel transcriptional potentiation of BETA2/NeuroD on the secretin gene promoter by the DNA-binding protein finb/RREB-1. *Mol. Cell. Biol.* **23**, 259–271 (2003).

16. E. Sanz, L. Yang, T. Su, D. R. Morris, G. S. McKnight, P. S. Amieux, Cell-type-specific isolation of ribosome-associated mRNA from complex tissues. *Proc. Natl. Acad. Sci. U.S.A.* **106**, 13939–13944 (2009).

17. Y. Liu, J. W. Lee, S. L. Ackerman, Mutations in the microtubule-associated protein 1A (Map1a) gene cause Purkinje cell degeneration. *J. Neurosci.* **35**, 4587–4598 (2015).

18. M. Ka, W.-Y. Kim, Microtubule-actin crosslinking factor 1 is required for dendritic arborization and axon outgrowth in the developing brain. *Mol. Neurobiol.* **53**, 6018–6032 (2016).

19. O. I. Kahn, P. Schätzle, D. van de Willige, R. P. Tas, F. W. Lindhout, S. Portegies, L. C. Kapitein, C. C. Hoogenraad, APC2 controls dendrite development by promoting microtubule dynamics. *Nat. Commun.* **9**, 2773 (2018).

20. D. Glick, S. Barth, K. F. Macleod, Autophagy: Cellular and molecular mechanisms. *J. Pathol.* **221**, 3–12 (2010).

21. C. Yang, X. Wang, Lysosome biogenesis: Regulation and functions. *J. Cell Biol.* **220,** e202102001 (2021).

22. T. Unno, M. Wakamori, M. Koike, Y. Uchiyama, K. Ishikawa, H. Kubota, T. Yoshida, H. Sasakawa, C. Peters, H. Mizusawa, K. Watase, Development of Purkinje cell degeneration in a knockin mouse model reveals lysosomal involvement in the pathogenesis of SCA6. *PNAS.* **109**, 17693–17698 (2012).

23. Y. R. Jo, H. R. Kim, S. Y. Jang, H. Go, M. Song, D. K. Park, Y. Oh, J. Jo, Y. K. Shin, S. J. Lee, S. Cheon, H. K. Lee, K. E. Lee, Y. H. Kim, H. T. Park, Potential neuron-autonomous Purkinje cell degeneration by 2′,3′-cyclic nucleotide 3′-phosphodiesterase promoter/Cre-mediated autophagy impairments. *FASEB J.* **35**, e21225 (2021).

24. M. Koike, M. Shibata, T. Sunabori, J. Yamaguchi, K. Sakimura, M. Komatsu, K. Tanaka, Y. Uchiyama, Purkinje cells are more vulnerable to the specific depletion of cathepsin D than to that of Atg7. *Am. J. Pathol.* **187**, 1586–1600 (2017).

25. S. S. Mann, J. A. Hammarback, Molecular characterization of light chain 3. A microtubule binding subunit of MAP1A and MAP1B. *J. Biol. Chem.* **269**, 11492–11497 (1994).

26. J. Scheel, R. Matteoni, T. Ludwig, B. Hoflack, T. E. Kreis, Microtubule depolymerization inhibits transport of cathepsin D from the Golgi apparatus to lysosomes. *J. Cell Sci.* **96**, 711–720 (1990).

27. E. Fass, E. Shvets, I. Degani, K. Hirschberg, Z. Elazar, Microtubules support production of starvation-induced autophagosomes but not their targeting and fusion with lysosomes. *J. Biol. Chem.* **281**, 36303–36316 (2006).

28. A. Aplin, T. Jasionowski, D. L. Tuttle, S. E. Lenk, W. A. Dunn, Cytoskeletal elements are required for the formation and maturation of autophagic vacuoles. *J. Cell. Physiol.* **152**, 458–466 (1992).

29. R. Köchl, X. W. Hu, E. Y. W. Chan, S. A. Tooze, Microtubules facilitate autophagosome formation and fusion of autophagosomes with endosomes. *Traffic* **7**, 129–145 (2006).

30. J. L. Webb, B. Ravikumar, D. C. Rubinsztein, Microtubule disruption inhibits autophagosome-lysosome fusion: Implications for studying the roles of aggresomes in polyglutamine diseases. *Int. J. Biochem. Cell Biol.* **36**, 2541–2550 (2004).

31. M. Götz, W. B. Huttner, The cell biology of neurogenesis. *Nat. Rev. Mol. Cell Biol.* **6**, 777–788 (2005).

32. E. W. Dent, S. L. Gupton, F. B. Gertler, The growth cone cytoskeleton in axon outgrowth and guidance. *Cold Spring Harb. Perspect. Biol.* **3**, a001800 (2011).

33. C. Conde, A. Cáceres, Microtubule assembly, organization and dynamics in axons and dendrites. *Nat. Rev. Neurosci.* **10**, 319–332 (2009).

34. G. Kerjan, J. G. Gleeson, Genetic mechanisms underlying abnormal neuronal migration in classical lissencephaly. *Trends Genet.* **23**, 623–630 (2007).

35. M. A. Stouffer, J. A. Golden, F. Francis, Neuronal migration disorders: Focus on the cytoskeleton and epilepsy. *Neurobiol. Dis.* **92**, 18–45 (2016).

36. G. Szebenyi, F. Bollati, M. Bisbal, S. Sheridan, L. Faas, R. Wray, S. Haferkamp, S. Nguyen, A. Caceres, S. T. Brady, Activity-driven dendritic remodeling requires microtubule-associated protein 1A. *Curr. Biol.* **15**, 1820–1826 (2005).

37. P.-Y. Shih, S.-P. Lee, Y.-K. Chen, Y.-P. Hsueh, Cortactin binding protein 2 increases microtubule stability and regulates dendritic arborization. *J. Cell Sci.* **32**, 1043–1055 (2014).

38. K. K. Gupta, C. Li, A. Duan, E. O. Alberico, O. V. Kim, M. S. Alber, H. V. Goodson, Mechanism for the catastrophe-promoting activity of the microtubule destabilizer Op18/stathmin. *PNAS.* **110**, 20449–20454 (2013).

39. E. Charbaut, P. A. Curmi, S. Ozon, S. Lachkar, V. Redeker, A. Sobel, Stathmin family proteins display specific molecular and tubulin binding properties. *J. Biol. Chem.* **276**, 16146–16154 (2001).

40. N. Ohkawa, K. Fujitani, E. Tokunaga, S. Furuya, K. Inokuchi, The microtubule destabilizer stathmin mediates the development of dendritic arbors in neuronal cells. *J. Cell Sci.* **120**, 1447–1456 (2007).

41. F. E. Poulain, S. Chauvin, R. Wehrlé, M. Desclaux, J. Mallet, G. Vodjdani, I. Dusart, A. Sobel, SCLIP is crucial for the formation and development of the Purkinje cell dendritic arbor. *J. Neurosci.* **28**, 7387–7398 (2008).

42. W. Liedtke, E. E. Leman, R. E. W. Fyffe, C. S. Raine, U. K. Schubart, Stathmin-deficient mice develop an age-dependent axonopathy of the central and peripheral nervous systems. *Am. J. Pathol.* **160**, 469–480 (2002).

43. Z. Melamed, J. López-Erauskin, M. W. Baughn, O. Zhang, K. Drenner, Y. Sun, F. Freyermuth, M. A. McMahon, M. S. Beccari, J. W. Artates, T. Ohkubo, M. Rodriguez, N. Lin, D. Wu, C. F. Bennett, F. Rigo, S. Da Cruz, J. Ravits, C. Lagier-Tourenne, D. W.

Cleveland, Premature polyadenylation-mediated loss of stathmin-2 is a hallmark of TDP-43-dependent neurodegeneration. *Nat. Neurosci.* **22**, 180–190 (2019).

44. J. D. Glass, Stathmin-2: Adding another piece to the puzzle of TDP-43 proteinopathies and neurodegeneration. *J. Clin. Investig.* **130**, 5677–5680 (2020).

45. O. A. Kent, M. Saha, E. Coyaud, H. E. Burston, N. Law, K. Dadson, S. Chen, E. M. Laurent, J. St-Germain, R. X. Sun, Y. Matsumoto, J. Cowen, A. Montgomery-Song, K. R. Brown, C. Ishak, J. La Rose, D. D. De Carvalho, H. H. He, B. Raught, F. Billia, P. Kannu, R. Rottapel, Haploinsufficiency of RREB1 causes a Noonan-like RASopathy via epigenetic reprogramming of RAS-MAPK pathway genes. *Nat. Commun.* **11**, 4673 (2020).

46. F. Lin, Z. V Wang, J. A. Hill, Seeing is believing. *Autophagy* **10**, 691–693 (2014).

47. K. J. Livak, T. D. Schmittgen, Analysis of relative gene expression data using real-time quantitative PCR and the $2^{-\Delta\Delta CT}$ method. *Methods* **25**, 402–408 (2001).

48. L. Texari, N. J. Spann, T. D. Troutman, M. Sakai, J. S. Seidman, S. Heinz, An optimized protocol for rapid, sensitive and robust on-bead ChIP-seq from primary cells. *STAR Protoc.* **2**, 100358 (2021).

49. S. Chen, Y. Zhou, Y. Chen, J. Gu, fastp: An ultra-fast all-in-one FASTQ preprocessor. *Bioinformatics* **34**, i884–i890 (2018).

50. B. Langmead, S. L. Salzberg, Fast gapped-read alignment with Bowtie 2. *Nat. Methods* **9**, 357–359 (2012).

51. Y. Liao, G. K. Smyth, W. Shi, featureCounts: An efficient general purpose program for assigning sequence reads to genomic features. *Bioinformatics* **30**, 923–930 (2014).

52. M. I. Love, W. Huber, S. Anders, Moderated estimation of fold change and dispersion for RNA-seq data with DESeq2. *Genome Biol.* **15**, 550 (2014).

53. S. X. Ge, D. Jung, R. Yao, ShinyGO: A graphical gene-set enrichment tool for animals and plants. *Bioinformatics* **36**, 2628–2629 (2020).

54. A. Tarasov, A. J. Vilella, E. Cuppen, I. J. Nijman, P. Prins, Sambamba: Fast processing of NGS alignment formats. *Bioinformatics* **31**, 2032–2034 (2015).

55. Y. Zhang, T. Liu, C. A. Meyer, J. Eeckhoute, D. S. Johnson, B. E. Bernstein, C. Nusbaum, R. M. Myers, M. Brown, W. Li, X. S. Liu, Model-based analysis of ChIP-seq (MACS). *Genome Biol.* **9**, R137 (2008).

56. S. Heinz, C. Benner, N. Spann, E. Bertolino, Y. C. Lin, P. Laslo, J. X. Cheng, C. Murre, H. Singh, C. K. Glass, Simple combinations of lineage-determining transcription factors prime cis-regulatory elements required for macrophage and B cell identities. *Mol. Cell* **38**, 576–589 (2010).

57. X. Ran, P. Zhou, K. Zhang, Autophagy plays an important role in stemness mediation and the novel dual function of EIG121 in both autophagy and stemness regulation of endometrial carcinoma JEC cells. *Int. J. Oncol.* **51**, 644–656 (2017).

58. L. Deng, J. Feng, R. R. Broaddus, The novel estrogen-induced gene EIG121 regulates autophagy and promotes cell survival under stress. *Cell Death Dis.* **1**, e32–e32 (2010).

59. C. J. Hong, J. Yeon, B. K. Yeo, H. Woo, H. An, W. Heo, K. Kim, S. Yu, Fas-apoptotic inhibitory molecule 2 localizes to the lysosome and facilitates autophagosome-lysosome fusion through the LC3 interaction region motif–dependent interaction with LC3. *FASEB J.* **34**, 161–179 (2020).

60. X. Roa-Mansergas, R. Fadó, M. Atari, J. F. Mir, H. Muley, D. Serra, N. Casals, CPT1C promotes human mesenchymal stem cells survival under glucose deprivation through the modulation of autophagy. *Sci. Rep.* **8**, 6997 (2018).

61. I. S. Stein, A. Gottfried, J. Zimmermann, G. Fischer von Mollard, TVP23interacts genetically with the yeast SNAREVTI1and functions in retrograde transport from the early endosome to the late Golgi. *Biochem. J.* **419**, 229–236 (2009).

62. W. Wang, Z. Xia, J.-C. Farré, S. Subramani, TRIM37 deficiency induces autophagy through deregulating the MTORC1-TFEB axis. *Autophagy* **14**, 1574–1585 (2018).

63. Y. Wen, R. G. Zhai, M. D. Kim, The role of autophagy in Nmnat-mediated protection against hypoxia-induced dendrite degeneration. *Mol. Cell. Neurosci.* **52**, 140–151 (2013).

64. A. K. H. Stavoe, S. E. Hill, D. H. Hall, D. A. Colón-Ramos, KIF1A/UNC-104 transports ATG-9 to regulate neurodevelopment and autophagy at synapses. *Dev. Cell* **38**, 171–185 (2016).

65. D. W. Lawrence, P. A. Willard, A. M. Cochran, E. C. Matchett, J. Kornbluth, Natural killer lytic-associated molecule (NKLAM): An E3 ubiquitin ligase with an integral role in innate immunity. *Front. Physiol.* **11**, 573372 (2020).

66. F. Nieto-Jacobo, D. Pasch, C. W. Basse, The mitochondrial Dnm1-Like fission component is required for lga2 -induced mitophagy but dispensable for starvation-induced mitophagy in ustilago maydis. *Eukaryot. Cell* **11**, 1154–1166 (2012).

67. M. Chen, Z. Chen, Y. Wang, Z. Tan, C. Zhu, Y. Li, Z. Han, L. Chen, R. Gao, L. Liu, Q. Chen, Mitophagy receptor FUNDC1 regulates mitochondrial dynamics and mitophagy. *Autophagy* **12**, 689–702 (2016).

68. C.-M. Xie, Y. Sun, The MTORC1-mediated autophagy is regulated by the FBXW7-SHOC2-RPTOR axis. *Autophagy* **15**, 1470–1472 (2019).

69. Y. Sun, W. Nie, B. Qiu, Q. Yang, H. Zhao, FBXW7 affects autophagy through MCL1 in oral squamous cell carcinoma. *Oral Dis.* **29**, 3259–3267 (2022).

70. L. Popineau, L. Morzyglod, N. Carré, M. Caüzac, P. Bossard, C. Prip-Buus, V. Lenoir, B. Ragazzon, V. Fauveau, L. Robert, S. Guilmeau, C. Postic, M. Komatsu, F. Canonne-Hergaux, H. Guillou, A.-F. Burnol, Novel Grb14-mediated cross talk between insulin and p62/Nrf2 pathways regulates liver lipogenesis and selective insulin resistance. *Mol. Cell. Biol.* **36**, 2168–2181 (2016).

71. E. Zavodszky, M. N. J. Seaman, K. Moreau, M. Jimenez-Sanchez, S. Y. Breusegem, M. E. Harbour, D. C. Rubinsztein, Mutation in VPS35 associated with Parkinson's disease impairs WASH complex association and inhibits autophagy. *Nat. Commun.* **5**, 3828 (2014).

72. K. Kato, Y. Oka, H. Muramatsu, F. F. Vasilev, T. Otomo, H. Oishi, Y. Kawano, H. Kidokoro, Y. Nakazawa, T. Ogi, Y. Takahashi, S. Saitoh, Biallelic VPS35L pathogenic variants cause 3C/Ritscher-Schinzel-like syndrome through dysfunction of retriever complex. *J. Med. Genet.* **57**, 245–253 (2020).

73. K. Jiang, M. Liu, G. Lin, B. Mao, W. Cheng, H. Liu, J. Gal, H. Zhu, Z. Yuan, W. Deng, Q. Liu, P. Gong, X. Bi, S. Meng, Tumor suppressor Spred2 interaction with LC3 promotes autophagosome maturation and induces autophagy-dependent cell death. *Oncotarget* **7**, 25652–25667 (2016).

74. M. Ullrich, B. Aßmus, A. M. Augustin, H. Häbich, M. Abeßer, J. Martin Machado, F. Werner, R. Erkens, A.-P. Arias-Loza, S. Umbenhauer, H. Wagner, P. M. Benz, A. Unger, W. A. Linke, S. Frantz, H. A. Baba, M. Kuhn, K. Schuh, SPRED2 deficiency elicits cardiac arrhythmias and premature death via impaired autophagy. *J. Mol. Cell. Cardiol.* **129**, 13–26 (2019).

75. C. Xie, S. Liu, B. Wu, Y. Zhao, B. Chen, J. Guo, S. Qiu, Y. Cao, miR-19 promotes cell proliferation, invasion, migration, and emt by inhibiting SPRED2-mediated autophagy in osteosarcoma cells. *Cell Transplant.* **29**, 963689720962460 (2020).

76. X. Liang, C. Wang, Y. Sun, W. Song, J. Lin, J. Li, X. Guan, p62/mTOR/LXRα pathway inhibits cholesterol efflux mediated by ABCA1 and ABCG1 during autophagy blockage. *Biochem. Biophys. Res. Commun.* **514**, 1093–1100 (2019).

77. S. L. Prosser, J. Tkach, L. Gheiratmand, J. Kim, B. Raught, C. G. Morrison, L. Pelletier, Aggresome assembly at the centrosome is driven by CP110–CEP97–CEP290 and centriolar satellites. *Nat. Cell Biol.* **24**, 483–496 (2022).

78. S. Imai, N. Okayama, M. Shimizu, M. Itoh, Increased intracellular calcium activates serum and glucocorticoid-inducible kinase 1 (SGK1) through a calmodulin-calcium calmodulin dependent kinase kinase pathway in Chinese hamster ovary cells. *Life Sci.* **72**, 2199–2209 (2003).

79. O. Borst, E.-M. Schmidt, P. Münzer, T. Schönberger, S. T. Towhid, M. Elvers, C. Leibrock, E. Schmid, A. Eylenstein, D. Kuhl, A. E. May, M. Gawaz, F. Lang, The serum- and glucocorticoid-inducible kinase 1 (SGK1) influences platelet calcium signaling and function by regulation of Orai1 expression in megakaryocytes. *Blood* **119**, 251–261 (2012).

80. T. R. H. Jouen-Tachoire, S. J. Tucker, P. Tammaro, Ion channels as convergence points in the pathology of pulmonary arterial hypertension. *Biochem. Soc. Trans.* **49**, 1855–1865 (2021).

81. H. V. McCue, R. D. Burgoyne, L. P. Haynes, Membrane targeting of the EF-hand containing calcium-sensing proteins CaBP7 and CaBP8. *Biochem. Biophys. Res. Commun.* **380**, 825–831 (2009).

82. F. Haeseleer, I. Sokal, C. L. M. J. Verlinde, H. Erdjument-Bromage, P. Tempst, A. N. Pronin, J. L. Benovic, R. N. Fariss, K. Palczewski, Five members of a novel Ca2+−binding protein (CABP) subfamily with similarity to calmodulin. *J. Biol. Chem.* **275**, 1247–1260 (2000).

83. P. H. Cheung, W. Luo, Y. Qiu, X. Zhang, K. Earley, P. Millirons, S. H. Lin, Structure and function of C-CAM1. The first immunoglobulin domain is required for intercellular adhesion. *J. Biol. Chem.* **268**, 24303–24310 (1993).

84. W. Luo, C. G. Wood, K. Earley, M.-C. Hung, S.-H. Lin, Suppression of tumorigenicity of breast cancer cells by an epithelial cell adhesion molecule (C-CAM1): The adhesion and growth suppression are mediated by different domains. *Oncogene* **14**, 1697–1704 (1997).

85. M. Terashima, M. Kobayashi, M. Motomiya, N. Inoue, T. Yoshida, H. Okano, N. Iwasaki, A. Minami, I. Matsuoka, Analysis of the expression and function of BRINP family genes during

neuronal differentiation in mouse embryonic stem cell-derived neural stem cells. *J. Neurosci. Res.* **88**, 1387–1393 (2010).

86. S. R. Berkowicz, T. J. Featherby, Z. Qu, A. Giousoh, N. A. Borg, J. I. Heng, J. C. Whisstock, P. I. Bird, Brinp1$^{-/-}$ mice exhibit autism-like behaviour, altered memory, hyperactivity and increased parvalbumin-positive cortical interneuron density. *Mol. Autism.* **7**, 22 (2016).

87. M. Hermann, R. Reumann, K. Schostak, D. Kement, M. Gelderblom, C. Bernreuther, R. Frischknecht, A. Schipanski, S. Marik, S. Krasemann, D. Sepulveda-Falla, M. Schweizer, T. Magnus, M. Glatzel, G. Galliciotti, Deficits in developmental neurogenesis and dendritic spine maturation in mice lacking the serine protease inhibitor neuroserpin. *Mol. Cell. Neurosci.* **102**, 103420 (2020).

88. R. Reumann, R. Vierk, L. Zhou, F. Gries, V. Kraus, J. Mienert, E. Romswinkel, F. Morellini, I. Ferrer, C. Nicolini, M. Fahnestock, G. Rune, M. Glatzel, G. Galliciotti, The serine protease inhibitor neuroserpin is required for normal synaptic plasticity and regulates learning and social behavior. *Learn. Mem.* **24**, 650–659 (2017).

89. J. Kashef, T. Diana, M. Oelgeschläger, I. Nazarenko, Expression of the tetraspanin family members Tspan3, Tspan4, Tspan5 and Tspan7 during *Xenopus laevis* embryonic development. *Gene Expr. Patterns* **13**, 1–11 (2013).

90. J. Xiao, R. Dai, L. Negyessy, C. Bergson, Calcyon, a novel partner of clathrin light chain, stimulates clathrin-mediated endocytosis. *J. Biol. Chem.* **281**, 15182–15193 (2006).

91. M. Kruusmägi, S. Zelenin, H. Brismar, L. Scott, Intracellular dynamics of calcyon, a neuron-specific vesicular protein. *Neuroreport* **18**, 1547–1551 (2007).

92. S.-E. Lee, S. Jeong, U. Lee, S. Chang, SGIP1α functions as a selective endocytic adaptor for the internalization of synaptotagmin 1 at synapses. *Mol. Brain* **12**, 41 (2019).

93. A. Uezu, A. Horiuchi, K. Kanda, N. Kikuchi, K. Umeda, K. Tsujita, S. Suetsugu, N. Araki, H. Yamamoto, T. Takenawa, H. Nakanishi, SGIP1α is an endocytic protein that directly interacts with phospholipids and Eps15. *J. Biol. Chem.* **282**, 26481–26489 (2007).

94. B. Ritter, S. Murphy, H. Dokainish, M. Girard, M. V. Gudheti, G. Kozlov, M. Halin, J. Philie, E. M. Jorgensen, K. Gehring, P. S. McPherson, NECAP 1 regulates AP-2 interactions to control vesicle size, number, and cargo during clathrin-mediated endocytosis. *PLoS Biol.* **11**, e1001670 (2013).

95. K. Goto, H. Kondo, Diacylglycerol kinase in the central nervous system—Molecular heterogeneity and gene expression. *Chem. Phys. Lipids* **98**, 109–117 (1999).

96. L. Wang, L. Zhu, C. C. Wang, The endoplasmic reticulum sulfhydryl oxidase Ero1β drives efficient oxidative protein folding with loose regulation. *Biochem. J.* **434**, 113–121 (2011).

97. M. Pagani, M. Fabbri, C. Benedetti, A. Fassio, S. Pilati, N. J. Bulleid, A. Cabibbo, R. Sitia, Endoplasmic reticulum oxidoreductin 1-Lβ (ERO1-Lβ), a human gene induced in the course of the unfolded protein response. *J. Biol. Chem.* **275**, 23685–23692 (2000).

98. M. W. Breuss, A. Nguyen, Q. Song, T. Nguyen, V. Stanley, K. N. James, D. Musaev, G. Chai, S. A. Wirth, P. Anzenberg, R. D. George, A. Johansen, S. Ali, M. Zia-ur-Rehman, T. Sultan, M. S. Zaki, J. G. Gleeson, Mutations in LNPK, encoding the endoplasmic reticulum junction stabilizer lunapark, cause a recessive neurodevelopmental syndrome. *Am. J. Hum. Genet.* **103**, 296–304 (2018).

99. A. Tsuchida, M. Ogiso, Y. Nakamura, M. Kiso, K. Furukawa, K. Furukawa, Molecular cloning and expression of human ST6GalNAc III: Restricted tissue distribution and substrate specificity. *J. Biochem.* **138**, 237–243 (2005).

100. Z. Chen, Z. G. Gulzar, C. A. St. Hill, B. Walcheck, J. D. Brooks, Increased expression of GCNT1 is associated with altered O-glycosylation of PSA, PAP, and MUC1 in human prostate cancers. *Prostate* **74**, 1059–1067 (2014).

101. E. Perkey, D. Maurice De Sousa, L. Carrington, J. Chung, A. Dils, D. Granadier, U. Koch, F. Radtke, B. Ludewig, B. R. Blazar, C. W. Siebel, T. V. Brennan, J. Nolz, N. Labrecque, I. Maillard, GCNT1-mediated O-glycosylation of the sialomucin CD43 is a sensitive indicator of notch signaling in activated T cells. *J. Immunol.* **204**, 1674–1688 (2020).

102. X. Li, J. Wang, W. Li, Y. Xu, D. Shao, Y. Xie, W. Xie, T. Kubota, H. Narimatsu, Y. Zhang, Characterization of ppGalNAc-T18, a member of the vertebrate-specific Y subfamily of UDP-N-acetyl-α-D-galactosamine:polypeptide N-acetylgalactosaminyltransferases. *Glycobiology* **22**, 602–615 (2012).

103. N. Naslavsky, J. McKenzie, N. Altan-Bonnet, D. Sheff, S. Caplan, EHD3 regulates early-endosome-to-Golgi transport and preserves Golgi morphology. *J. Cell Sci.* **122**, 389–400 (2009).

104. K. Bahl, S. Xie, G. Spagnol, P. Sorgen, N. Naslavsky, S. Caplan, EHD3 protein is required for tubular recycling endosome stabilization, and an asparagine-glutamic acid residue pair within its Eps15 homology (EH) domain dictates its selective binding to NPF peptides. *J. Biol. Chem.* **291**, 13465–13478 (2016).

105. T. K. Bjarnadóttir, R. Fredriksson, H. B. Schiöth, The gene repertoire and the common evolutionary history of glutamate, pheromone (V2R), taste(1) and other related G protein-coupled receptors. *Gene* **362**, 70–84 (2005).

106. J. Sheng, J. Xu, K. Geng, D. Liu, Sema6D regulates zebrafish vascular patterning and motor neuronal axon growth in spinal cord. *Front Mol Neurosci.* **15**, 854556 (2022).

107. A. Garbouchian, A. C. Montgomery, S. P. Gilbert, M. Bentley, KAP is the neuronal organelle adaptor for kinesin-2 KIF3AB and KIF3AC. *Mol. Biol. Cell* **33**, ar133 (2022).

108. H. Yamazaki, T. Nakata, Y. Okada, N. Hirokawa, Cloning and characterization of KAP3: A novel kinesin superfamily-associated protein of KIF3A/3B (microtubule/axonal transport/motor protein). *Cell Biol.* **93**, 8443–8448 (1996).

109. M. A. Felmlee, R. S. Jones, V. Rodriguez-Cruz, K. E. Follman, M. E. Morris, Monocarboxylate transporters (SLC16): Function, regulation, and role in health and disease. *Pharmacol. Rev.* **72**, 466–485 (2020).

110. S. Poliak, L. Gollan, R. Martinez, A. Custer, S. Einheber, J. L. Salzer, J. S. Trimmer, P. Shrager, E. Peles, Caspr2, a new member of the neurexin superfamily, is localized at the

juxtaparanodes of myelinated axons and associates with k channels. *Neuron* **24**, 1037–1047 (1999).

111. F. St George-Hyslop, M. Haneklaus, T. Kivisild, F. J. Livesey, Loss of CNTNAP2 alters human cortical excitatory neuron differentiation and neural network development. *Biol. Psychiatry* **94**, 780–791 (2023).

112. M. Poot, Connecting the CNTNAP2 networks with neurodevelopmental disorders. *Mol Syndromol.* **6**, 7–22 (2015).

113. M. Gaitanou, K. Segklia, R. Matsas, Cend1, a story with many tales: From regulation of cell cycle progression/exit of neural stem cells to brain structure and function. *Stem Cells Int.* **2019**, 1–16 (**2019**).

114. A. I. R Spanjer, H. A. Baarsma, L. M. Oostenbrink, S. R. Jansen, C. C. Kuipers, M. Lindner, D. S. Postma, H. Meurs, I. H. Heijink, R. Gosens, M. Königshoff, A. M. Fachkliniken, TGF-b-induced profibrotic signaling is regulated in part by the WNT receptor Frizzled-8. *J (Basel).* **30**, 1823–1835 (2016).

115. K. Itoh, J. Jacob, S. Y. Sokol, A role for xenopus Frizzled 8 in dorsal development. *Mech. Dev.* **74**, 145–157 (1998).

116. M. A. Deardorff, C. Tan, L. J. Conrad, P. S. Klein, Frizzled-8 is expressed in the Spemann organizer and plays a role in early morphogenesis. *Development* **125**, 2687–2700 (1998).

117. D. J. Gillooly, A. Simonsen, H. Stenmark, Cellular functions of phosphatidylinositol 3-phosphate and FYVE domain proteins. *Biochem. J.* **355**, 249–258 (2001).

118. N. J. Blunsom, S. Cockcroft, CDP-diacylglycerol synthases (CDS): Gateway to phosphatidylinositol and cardiolipin synthesis. *Front. Cell Dev. Biol.* **8**, 63 (2020).

119. P.-L. Hsu, J. Jou, S.-J. Tsai, TYRO3: A potential therapeutic target in cancer. *Exp. Biol. Med.* **244**, 83–99 (2019).

120. S. Chauvin, A. Sobel, Neuronal stathmins: A family of phosphoproteins cooperating for neuronal development, plasticity and regeneration. *Prog. Neurobiol.* **126**, 1–18 (2015).

121. V. C. Foletta, N. Moussi, P. D. Sarmiere, J. R. Bamburg, O. Bernard, LIM kinase 1, a key regulator of actin dynamics, is widely expressed in embryonic and adult tissues. *Exp. Cell Res.* **294**, 392–405 (2004).

122. D. C. Muñoz-Lasso, C. Romá-Mateo, F. V. Pallardó, P. Gonzalez-Cabo, Much more than a Scaffold: Cytoskeletal proteins in neurological disorders. *Cell* **9**, 358 (2020).

123. C. Y. Lin, H. L. Lai, H. M. Chen, J. J. Siew, C. Te Hsiao, H. C. Chang, K. S. Liao, S. C. Tsai, C. Y. Wu, K. Kitajima, C. Sato, K. H. Khoo, Y. Chern, Functional roles of ST8SIA3-mediated sialylation of striatal dopamine D2 and adenosine A2A receptors. *Transl. Psychiatry* **2019**. **9**, 1–13 (**2019**).

124. R.-B. Huang, D. Cheng, S.-M. Liao, B. Lu, Q.-Y. Wang, N.-Z. Xie, F. A. Troy II, G.-P. Zhou, The intrinsic relationship between structure and function of the sialyltransferase ST8Sia family members. *Curr. Top. Med. Chem.* **17**, 2359–2369 (2017).

125. J. Liu, X. Zheng, X. Pang, L. Li, J. Wang, C. Yang, G. Du, Ganglioside GD3 synthase (GD3S), a novel cancer drug target. *Acta Pharm. Sin.* **8**, 713–720 (2018).

126. E. R. Sturgill, K. Aoki, P. Hh Lopez, D. Colacurcio, K. Vajn, I. Lorenzini, S. Majić, W. H. Yang, M. Heffer, M. Tiemeyer, J. D. Marth, R. L. Schnaar, Biosynthesis of the major brain gangliosides GD1a and GT1b. *Glycobiology* **22**, 1289–1301 (2012).

127. S. W. Yoo, M. G. Motari, K. Susuki, J. Prendergast, A. Mountney, A. Hurtado, R. L. Schnaar, Sialylation regulates brain structure and function. *FASEB J.* **29**, 3040–3053 (2015).

128. N. W. Shworak, J. Liu, L. M. Petros, L. Zhang, M. Kobayashi, N. G. Copeland, N. A. Jenkins, R. D. Rosenberg, Multiple isoforms of heparan sulfate D-glucosaminyl 3-O-sulfotransferase: Isolation, characterization, and expression of human cdnas and identification of distinct genomic loci. *J. Biol. Chem.* **274**, 5170–5184 (1999).

129. H. Ota, S. Nishihara, Regulation of 3-O-sulfation of heparan sulfate during transition from the naïve to the primed state in mouse embryonic stem cells. *Methods Mol. Biol.* **2303**, 443–452 (2022).

130. N. Kaempf, G. Kochlamazashvili, D. Puchkov, T. Maritzen, S. M. Bajjalieh, N. L. Kononenko, V. Haucke, Overlapping functions of stonin 2 and SV2 in sorting of the calcium sensor synaptotagmin 1 to synaptic vesicles. *Proc. Natl. Acad. Sci. U.S.A.* **112**, 7297–7302 (2015).

131. A. Nowack, J. Yao, K. L. Custer, S. M. Bajjalieh, SV2 regulates neurotransmitter release via multiple mechanisms. *Am. J. Physiol. Cell Physiol.* **299**, 960–967 (2010).

132. V. Rossi, R. Picco, M. Vacca, M. D'Esposito, M. D'Urso, T. Galli, F. Filippini, VAMP subfamilies identified by specific R-SNARE motifs. *Biol. Cell* **96**, 251–256 (2004).

133. Y. Wu, H. Matsui, K. Tomizawa, Amphiphysin I and regulation of synaptic vesicle endocytosis. *Acta Med. Okayama* **63**, 305–323 (2009).

134. C. David, P. S. McPherson, O. Mundigl, P. De Camilli, A role of amphiphysin in synaptic vesicle endocytosis suggested by its binding to dynamin in nerve terminals. *PNAS.* **93**, 331–335 (1996).

135. F. Antonucci, I. Corradini, G. Fossati, R. Tomasoni, E. Menna, M. Matteoli, SNAP-25, a known presynaptic protein with emerging postsynaptic functions. *Front Synaptic Neurosci.* **8**, 187218 (2016).

136. S. Sugita, R. Janz, T. C. Südhof, Synaptogyrins regulate Ca2+−dependent exocytosis in PC12 Cells. *J. Biol. Chem.* **274**, 18893–18901 (1999).

137. R. Mohrmann, H. de Wit, E. Connell, P. S. Pinheiro, C. Leese, D. Bruns, B. Davletov, M. Verhage, J. B. Sørensen, Synaptotagmin interaction with SNAP-25 governs vesicle docking, priming, and fusion triggering. *J. Neurosci.* **33**, 14417–14430 (2013).

138. R. G. Whittaker, D. N. Herrmann, B. Bansagi, B. A. S. Hasan, R. M. Lofra, E. L. Logigian, J. E. Sowden, J. L. Almodovar, J. T. Littleton, S. Zuchner, R. Horvath, H. Lochmüller, Electrophysiologic features of SYT2 mutations causing a treatable neuromuscular syndrome. *Neurology* **85**, 1964–1971 (2015).
